# A sterol panel for rare lipid disorders: sitosterolemia, cerebrotendinous xanthomatosis, and Smith-Lemli-Opitz syndrome

**DOI:** 10.1016/j.jlr.2024.100698

**Published:** 2024-11-19

**Authors:** Alexander Bauer Westbye, Lili L. Dizdarevic, Sandra R. Dahl, Emil Andreas Asprusten, Yngve Thomas Bliksrud, Anita Lövgren Sandblom, Ulf Diczfalusy, Per M. Thorsby, Kjetil Retterstøl

**Affiliations:** 1 Hormone Laboratory, Department of Medical Biochemistry, Oslo University Hospital, Aker, Oslo, Norway; 2 Biochemical Endocrinology and Metabolism Research Group, Oslo University Hospital, Aker, Oslo, Norway; 3 Lipid Clinic, Oslo University Hospital, Aker, Oslo, Norway; 4 Norwegian National Unit for Diagnostics of Congenital Metabolic Disorders, Department of Medical Biochemistry, Oslo University Hospital, Rikshospitalet, Oslo, Norway; 5 Metabolic Molecular Biology Research Group, Oslo University Hospital, Rikshospitalet, Oslo, Norway; 6 Division of Clinical Chemistry, Department of Laboratory Medicine, Karolinska Institutet, Stockholm, Sweden; 7 Clinical Chemistry, Karolinska University Hospital, Stockholm, Sweden; 8 Institute of Clinical Medicine, University of Oslo, Oslo, Norway; 9 Department of Nutrition, Institute of Basic Medical Sciences, University of Oslo, Oslo, Norway

**Keywords:** sitosterol, campesterol, stigmasterol, 7-dehydrocholesterol, cholestanol, LC-MS/MS

## Abstract

Disease-specific sterols accumulate in the blood of patients with several rare lipid disorders. Biochemical measurement of these sterols is important for correct diagnosis and sometimes monitoring of treatment. Existing methods to measure sterols in blood, particularly plant sterols, are often laborious and time consuming. Partly as a result, clinical access to sterol measurements is limited in many parts of the world. A simple and rapid method to extract free sterols from human serum and quantitate their concentration using isotope-dilution liquid chromatography-tandem mass spectrometry (LC-MS/MS) without derivatization was developed. The method was designed to be compatible with routine workflows (e.g., 96-well format) in a clinical lab and extensively validated. Serum from at least 125 controls were analyzed and used to estimate the upper reference limits for sitosterol, campesterol, stigmasterol, desmosterol, 7-dehydrocholesterol (7DHC), lathosterol, and cholestanol. Serum from patients with the rare lipid disorders sitosterolemia (n = 7), Smith-Lemli-Opitz syndrome (SLOS; n = 1), and cerebrotendinous xanthomatosis (CTX; n = 1) were analyzed. All seven sitosterolemia patients had greatly elevated levels of free plant sterols (sitosterol, campesterol, and stigmasterol) compared to the controls. The SLOS serum contained massively increased concentrations of 7DHC. CTX serum contained greatly increased concentrations of cholestanol, as well as 7DHC and lathosterol. Spiking experiments indicated that the method is likely also useful for the diagnosis of desmosterolosis and lathosterolosis. The reported method is a relatively simple and fast LC-MS/MS method capable of quantitating diagnostically important sterols and differentiated patients with three rare lipid disorders from controls.

## Graphical abstract

**Figure:**
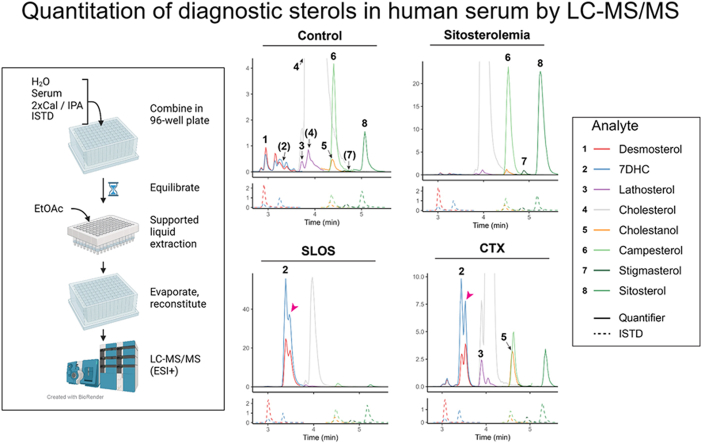
Quantitation of diagnostic sterols (free, nonesterified) in human serum using a simple and fast LC-MS/MS method. Sitosterolemia, Smith-Lemli-Opitz syndrome (SLOS), and cerebrotendinous xanthomatosis (CTX) patients had greatly increased concentrations compared to controls.

Disturbances in the biosynthesis, metabolism, or transport of sterols underlie several rare lipid disorders, including sitosterolemia (phytosterolemia), Smith-Lemli-Opitz syndrome (SLOS), cerebrotendinous xanthomatosis (CTX), desmosterolosis, and lathosterolosis (1, 2, 3). Affected patients typically accumulate specific sterols or stanols (saturated sterols; hereon included in the term “sterols”) to high concentrations in plasma and tissues. Measurement of these biomarkers in plasma or serum are important for diagnosis and sometimes monitoring of treatment (e.g., sitosterolemia and CTX) (1, 2, 3). Clinical access to measurement methods of noncholesterol sterols is limited, and especially plant sterol analyses are scarce.

Sterols are highly hydrophobic molecules and are mainly transported in lipoprotein particles in blood. Most (if not all) sterols, including cholesterol, plant sterols (elevated in sitosterolemia), 7-dehydrocholesterol (7DHC; SLOS), and cholestanol (CTX) are transported in the blood both as free (nonesterified) sterols and as steryl-esters (sterols esterified with a fatty acid) (4, 5, 6, 7, 8, 9) (supplemental Table S1). Most methods measure the total concentration (combined free and esterified sterols), and this requires complex and labor intensive sample preparations with multiple manual steps before mass spectrometry detection. Saponification (base hydrolysis) at elevated temperatures, liquid-liquid extraction (often using hazardous chlorinated solvents), and derivatization are commonly employed (6, 9, 10, 11, 12, 13). However, several studies indicate that both the free and esterified concentrations of the relevant sterol(s) are increased by disease and that the percentage of free sterol concentration (to the total concentration) is relatively consistent at 25%–45% depending on the sterol, at least on a group level (summarized in supplemental Table S1). If measurement of free sterols is sufficient for biochemical diagnosis and possibly monitoring of at least some sterol disorders, we suspected that this could be performed using a much simpler analytical workflow.

Here, we report a simple and fast sterol-panel suitable for workflows in clinical routine laboratories employing liquid chromatography-tandem mass spectrometry (LC-MS/MS) (Fig. 1): The method measures the free concentration of seven diagnostic sterols and employs 96-well format supported-liquid extraction (SLE) and absolute quantitation using isotope-dilution. Upper reference limits for free sterols in nonaffected individuals were estimated. The method clearly distinguished previously diagnosed sitosterolemia, SLOS, and CTX patients from controls.

**Fig. 1:**
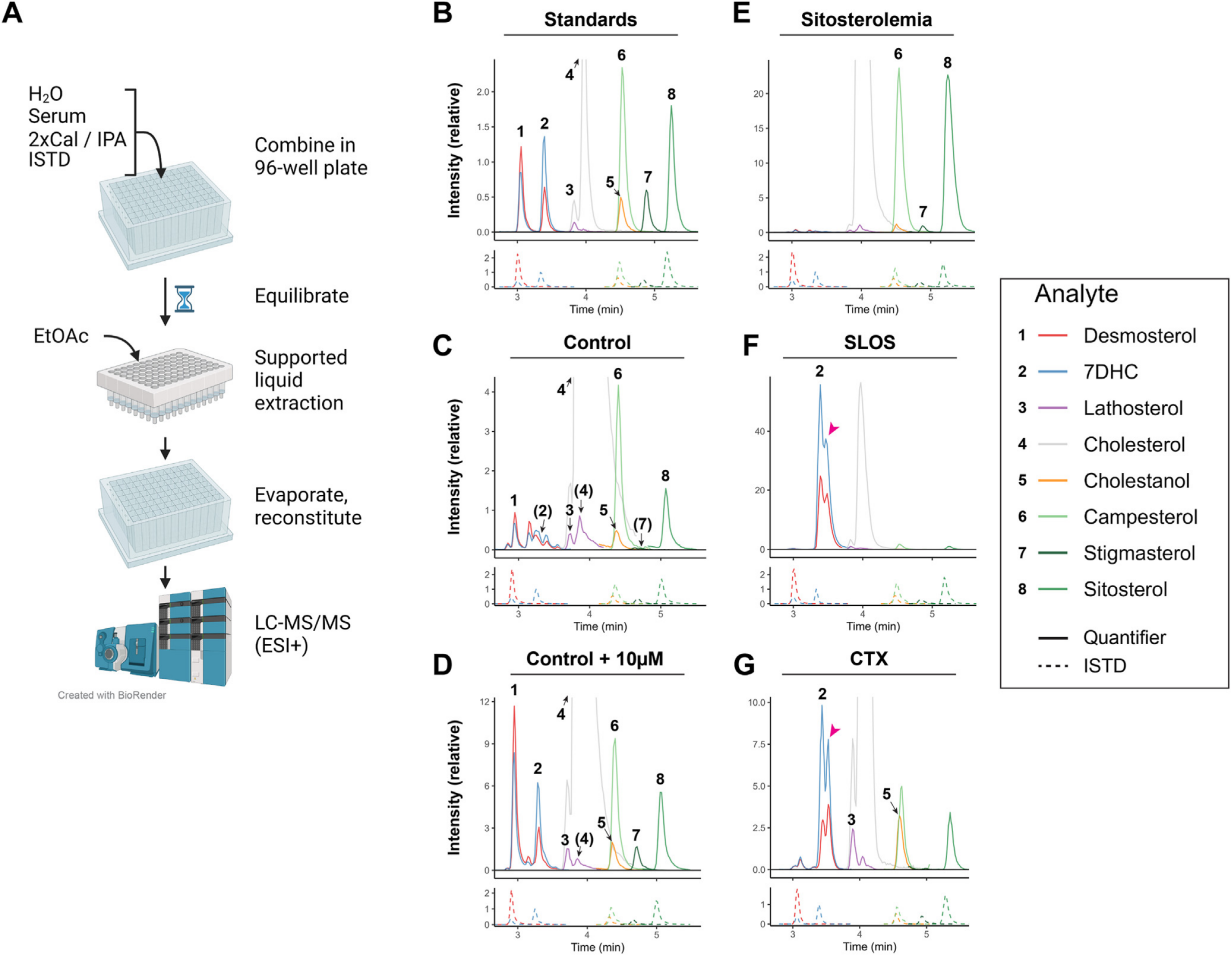
Measurement of free sterols in human serum. Overview of method (A) and qualitative visualization of sterol profiles (extracted ion chromatograms; XICs) (B to G). A: Serum is combined with water, isopropanol (IPA), and internal standard (ISTD) stock in a 96-well plate. Calibrators are prepared using 2-fold concentrated calibrator stocks (2xCal; in IPA) added to lipoprotein-depleted serum. After equilibration, the mixture is transferred to a supported liquid extraction plate and extracted with ethyl acetate (EtOAc). Extract is evaporated, reconstituted, and injected on LC-MS/MS employing electrospray ionization to measure free (non-acyl conjugated) sterols. B: Standards in lipoprotein-depleted serum (Calibration standard 6). C: Control serum pool from presumed nonaffected patients. D: Control pool spiked with 10 μM of each sterol (excluding cholesterol). E: Sitosterolemia patient serum (pool). F: Smith-Lemli-Opitz Syndrome (SLOS) patient serum. G: Cerebrotendinous Xanthomatosis (CTX) patient serum. Numbers indicate analyte (parenthesis indicate absence or the cholesterol-signal present in the lathosterol XIC), and either all analytes (B to D) or analytes elevated compared to control (E to G) are indicated. Magenta arrowhead indicate presence of elevated unidentified sterol (presumed to be 8DHC). Solid XICs are from the quantitation-transitions and dashed-lines the ISTDs. For increased visual clarity, the XICs were trimmed around the known retention time and were normalized to the max. peak height of the 7DHC-ISTD. Created in BioRender (https://BioRender.com/h87p026).

## Materials and methods

A detailed version of the materials and methods section is presented in the supplemental material.

### Ethical and data protection approvals

The study has been approved by The Regional Committee of Medical and Health Research Ethics for South-Eastern Norway (ID numbers: 521264 and 687927) and by the Norwegian Data Protection Official at Oslo University Hospital (Norwegian: “Oslo universitetssykehus”, abbreviated OUS; ID numbers 22/25,275 and 23/27,629). This work abides by the Declaration of Helsinki principles.

### Patient samples

Analyzed serum originated from several sources: participants in the on-going study “Sitosterolemia in Norway”, including seven sitosterolemia patients (previously verified by genetic tests) from five unrelated families referred to the Lipid Clinic at OUS. One SLOS and one CTX patient (genetically verified; SLOS was also verified biochemically) from the Norwegian National Unit for Diagnostics of Congenital Metabolic Disorders (OUS). Control samples from presumed nonaffected/healthy (in respect to rare sterol disorders) individuals from leftover, anonymized patient serum samples received as part of routine diagnostic testing at the Hormone Laboratory (OUS).

### Simple and rapid extraction of free sterols from human serum

In brief, 50 μl serum was mixed with deuterated internal standards and extracted using 96-well format supported liquid extraction (Biotage Isolute SLE+) with ethyl acetate (Fig. 1A).

Calibrators were prepared in lipoprotein deficient serum from human plasma (Sigma-Aldrich).

### Quantitation of free sterols by LC-MS/MS

A LC-MS/MS method to quantitate free sterols in the extract was developed. In brief, sterols (not derivatized) were separated on a phenyl hexyl column (Phenomenex Kinetex) using a H_2_O:acetonitrile gradient and ionized by electrospray ionization in positive mode (ESI+). Sterols were measured using scheduled multiple reaction monitoring on a Sciex QTRAP 6500+.

### Method validation

The method was extensively validated. We investigated quantitation limits, linearity, imprecision, accuracy (“spike and recovery”), matrix effects, and carryover.

Measurements were compared to other clinical methods. Sitosterol and campesterol concentrations were compared to a GC-MS method at Karolinska Institutet (KI) (14). 7DHC and cholestanol concentrations were compared to “European Research Network for Evaluation and Improvement of Screening, Diagnosis and Treatment of Inherited Disorders of Metabolism” (ERNDIM) control samples. Samples above the method upper limit of quantitation were diluted in lipoprotein-deficient serum.

### Estimated upper reference limits for clinical guidance

Upper reference values for clinical guidance were estimated from a control group of at least 125 samples by calculating the upper limit of a 95% one-sided reference interval for each analyte. For stigmasterol and 7DHC, the upper limit was set to be below method lower limit of quantitation (mLLOQ).

### Software and data analysis

The LC-MS/MS was controlled by Sciex Analyst and quantitation performed using Sciex OS Analytics. All peaks were manually inspected. Data handling was performed using Microsoft Excel (2016) and R (v 4.3.0) using RStudio (v 2023.09.01) (15, 16).

## Results

### Development and validation of a rapid sample extraction and LC-MS/MS method for measurement of free sterols in human serum

A simple and rapid sample extraction procedure using 96-well format SLE and a relatively fast (<10 min) ESI+ LC-MS/MS method to quantitate sterols in human serum was developed (Fig. 1A). Extraction requires no chlorinated solvents, no saponification, and no derivatization. The method measures seven diagnostically relevant sterols (and cholesterol) and incorporates six isotope-labeled internal standards for absolute quantiation of sitosterol, campesterol, stigmasterol, desmosterol, 7DHC and cholestanol, and semiquantitation of lathosterol, in relevant patient serum (Fig 1B–G, supplemental Table S1; additional details in Supplemental Material).

Desmosterol, cholestanol, campesterol, and sitosterol were baseline-separated from other compounds and present in control serum (“healthy” individuals) at quantities allowing reliable and accurate quantitation (Fig. 1B, C, supplemental Fig. S1, supplemental Tables S6–S8). 7DHC, lathosterol and stigmasterol were either not present or not consistently fully separated from interferences for accurate and/or reliable quantitation in control serum (Fig. 1C, supplemental Figs. S4C, S5A, and S6C) but were markedly elevated and could be quantitated after spiking (supplemental Fig. S1, supplemental Tables S6–S8). Importantly, these sterols were markedly increased in the relevant patient groups (Fig. 1E–G) as reported below.

### Free sterol concentrations in nonaffected individuals and estimated upper reference limits

Serum from 126 presumed-healthy (with respect to rare lipid disorders; routine samples received for three unrelated biochemical tests) were analyzed to establish estimates of sterol concentrations in “healthy” individuals and upper reference limits. No gross differences between the three groups were evident (supplemental Fig. S2A), and the groups were therefore combined (supplemental Fig. S2B). After removal of maximum one outlier per sterol, summary statistics and the estimated upper limits for the control population were calculated (Table 1).

**Table 1:** Free sterol concentrations in control samples

Analyte (n)	Range^a^	Mean^a^	SD^a^	Median^a^	Q1^a^	Q3^a^	Upper reference limit (90% CI)^a^
Desmosterol (125)	0.06–2.3	0.53	0.29	0.46	0.33	0.65	0.9 (0.9–1.2)
7DHC (126)^b^	< 5.7	NA	NA	NA	NA	NA	< 5.7 (NA)
Lathosterol (126)^c^	0.06–8.11	1.88	1.27	1.56	1.02	2.5	See footnote c.
Cholestanol (125)	0.71–5.99	2.19	0.9	2.06	1.61	2.49	3.9 (3.5–4.8)
Campesterol (126)	0.12–16.52	4.21	2.82	3.58	2.14	5.54	9.9 (7.7–12.1)
Stigmasterol (126)^b^	< 1.5	NA	NA	NA	NA	NA	< 1.5 (NA)
Sitosterol (125)	0.12–7.84	2.56	1.62	2.18	1.49	3.38	6.1 (5.2–6.6)

CI, confidence interval from bootstrapping; 7DHC, 7-dehydrocholesterol; ISTD, internal standard; mLLOQ, method lower limit of quantitation; NA, not available; SD, standard deviation; Q1, first quartile; Q3, third quartile.

All concentration values are in μmoles/L (μM). Concentrations can be converted from μM to mg/L by multiplying concentrations with the conversion factors 0.385 (desmosterol and 7DHC), 0.387 (lathosterol), 0.389 (cholestanol), 0.401 (campesterol), 0.413 (stigmasterol), or 0.415 (sitosterol).

a Statistical parameters calculated after removal of max. one outlier per sterol and imputation of any values below the quantitative limits. For lathosterol, some values were calculated after extrapolation of the calibrator curve.

b All stigmasterol and 7DHC concentrations in control samples were below mLLOQ.

c Lathosterol is only semi-quantitative and lacks analyte-specific ISTD. The calculated upper limit from our dataset is 4.1 (90% CI: 3.5–4.4) μM.

### Comparison of plant sterol measurements to a GC-MS method

**Fig. 2:**
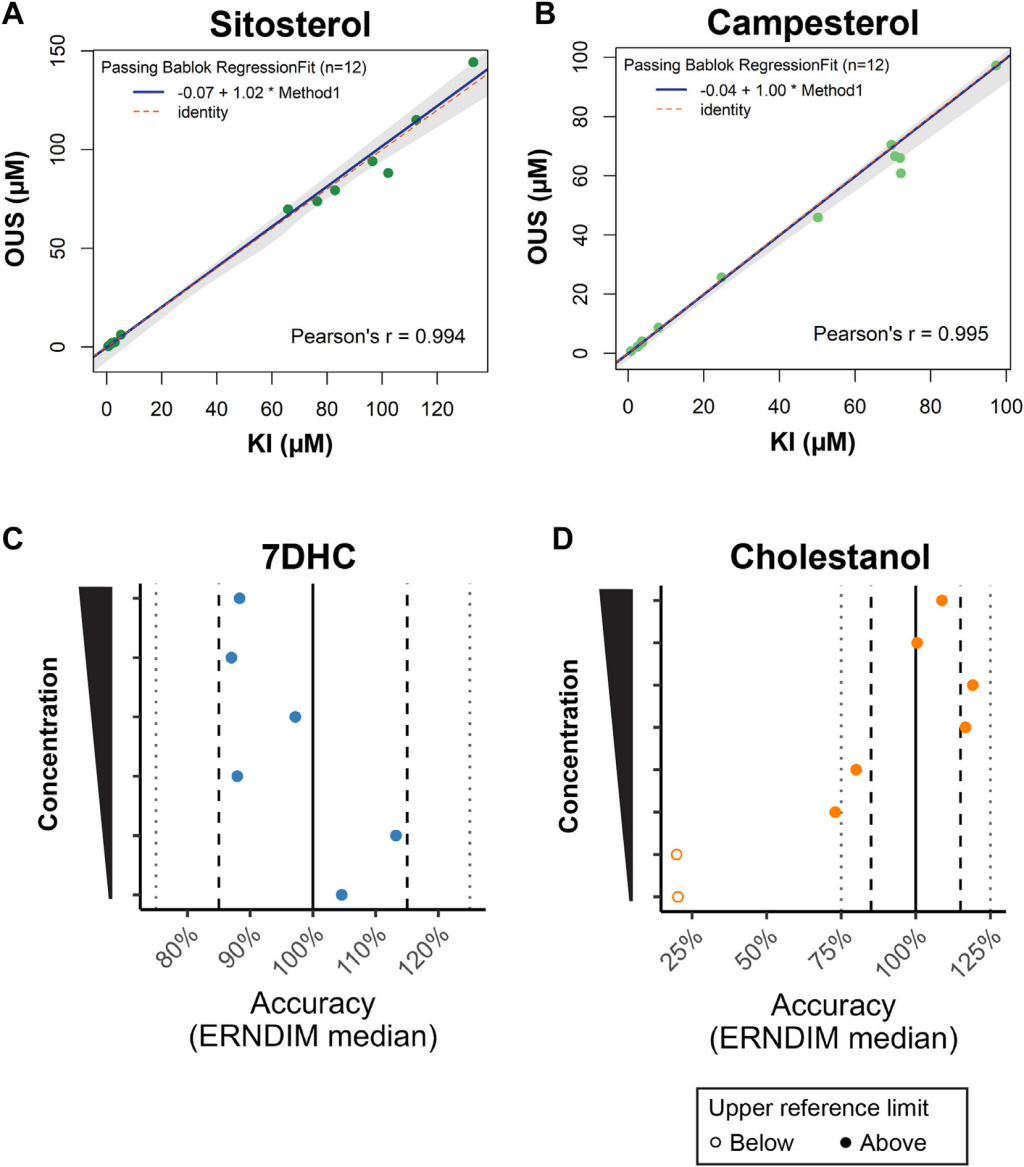
Method comparisons. Comparison of measured sitosterol (A), campesterol (B), 7DHC (C), and cholestanol (D) concentrations. A and B: Passing-Bablok comparison of results measured by reported method (OUS) to free plant sterol concentrations measured by Karolinska Institutet (KI). C and D: Comparison to reported consensus concentrations (median) for ERNDIM external quality assurance samples, arranged based on the consensus concentration. For D, filled circles indicate concentration values above the upper reference limit of our method (see Table 1). The ERNDIM consensus values are confidential and therefore not reported.

Measured free sitosterol and campesterol concentrations with the reported method were compared to free and total concentrations measured by a GC-MS method in clinical use at KI: Free plant sterols concentrations correlated well (Pearson’s r ≥ 0.99) and measured concentrations were very similar (Fig. 2A, B, supplemental Fig. S3A, B).

As expected, free (OUS) and total (KI) plant sterol concentrations also correlated well (Pearson’s r ≥ 0.98) and were linearly related with a regression slope of approx. 0.3 for both sterols (supplemental Fig. S3C, D).

### Investigation of 7DHC and cholestanol quantitation accuracy using ERNDIM samples

We investigated the methods ability to quantitate 7DHC and cholestanol using ERNDIM-distributed external quality assurance (EQA) and Internal Quality Control System Special Assays in Serum (SAS) samples. Normal serum contains very low concentrations of 7DHC but readily measurable concentrations of free cholestanol (Table 1 and supplemental Table S1). To produce samples with elevated sterol concentrations, ERNDIM spikes the SAS (and most likely EQA) samples with sterol standards, according to provided documentation. Samples spiked with a high concentrations of sterols will therefore predominantly contain free sterols. Our measured free sterol concentration should therefore be comparable to other measurements (whether total or free sterols) and give an indication of the quantitative accuracy.

Two SAS and eight EQA samples were analyzed. For 7DHC, the SAS samples were measured to contain 18.8 and 85.5 μM, concentrations far above our normal range (likely far below 5.7 μM, Table 1) and similar to the target concentrations (118% and 107% accuracy; supplemental Table S9). Six (of eight) EQA samples contained sufficient 7DHC for reliable quantitation (spanning concentrations of approx. 1.5- to 25-times our maximal upper reference limit). Our measurements were comparable (87%–113% accuracy) to the ERNDIM-reported consensus concentrations (Fig. 2C and supplemental Fig. S3G).

Two EQA samples were measured to contain free cholestanol within our reference range (≤3.9 μM) and were likely unadulterated (not spiked with cholestanol). Our concentrations were approx. 20% of the consensus concentration (Fig. 2D and supplemental Fig. S3H), values slightly lower than the expected 25%–38% based on the reported percentage of free cholestanol (7, 8). For the other six EQA samples, measured free cholestanol was approx. 3.5- to 25-times our upper reference limit. For these, our measurements were generally comparable (ranging from 73% to 119% accuracy; Fig. 2D and supplemental Fig. S3H) to reported consensus concentrations. For the two SAS samples, free cholestanol was measured to 21 and 66 μM (approx. 4- and 16-times our upper reference limit, respectively) and corresponded to 75% and 92% of the expected values (supplemental Table S9), respectively.

To summarize, our own spike-and-recovery experiments (supplemental Fig. S1, supplemental Table S8), comparison with a GC-MS method (Fig 2A, B) and analysis of the spiked ERNDIM-samples (Fig. 2C, D) indicate that our method can quantitate free sterols (except lathosterol) with a relatively high accuracy.

### Sitosterolemia patients: markedly increased free plant sterol concentrations

Patients with sitosterolemia accumulate plant sterols, especially sitosterol, in their serum. Typically, a laborious sample preparation involving saponification and liquid-liquid extraction with chlorinated solvents are used to extract the total (free and esterified) sterol fraction. This method complexity and the resultant high cost is likely an important reasons for the very low availability of clinical sitosterol analyses and a barricade to improved diagnosis of sitosterolemia.

We investigated whether sitosterolemia patients could be discriminated from controls by measuring free plant sterol concentrations in serum from one untreated and six sitosterolemia patients on treatment to lower their plant sterol concentrations.

**Fig. 3:**
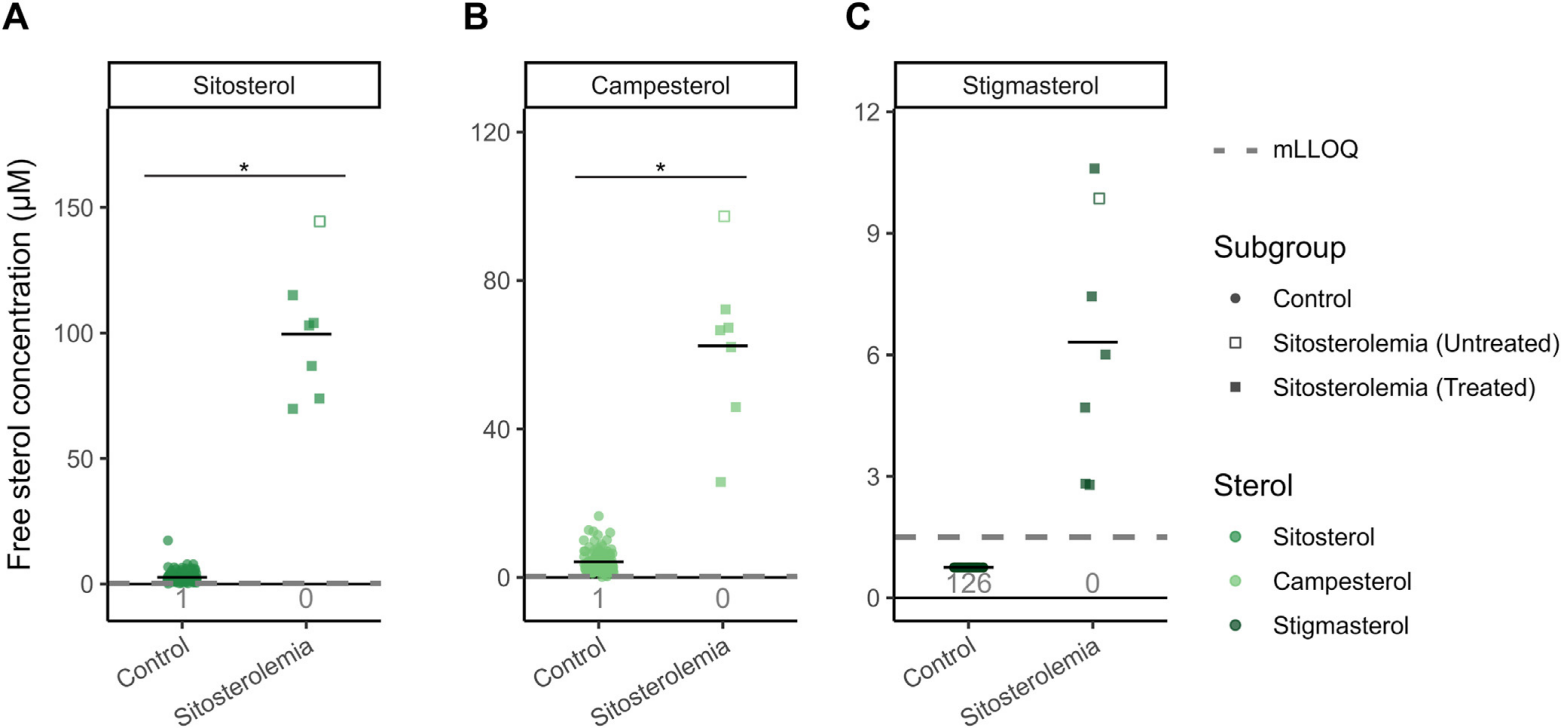
Greatly increased free plant sterol concentrations in sitosterolemia. Measured concentrations of free plant sterols in seven sitosterolemia patients (six on treatment to lower plant sterols) and 126 controls. A: Sitosterol. B: Campesterol. C: Stigmasterol. Points show individual values, and black bars indicate group median. The dashed gray lines indicate the method lower limit of quantitation (mLLOQ) and the gray text the no. of samples below the mLLOQ. Asterisk (∗) indicates statistically significant Mann-Whitney U test results, *P*-value < 0.001.

The plant sterol extracted ion chromatograms from all sitosterolemia patients were qualitatively markedly different from the controls. All seven patients had greatly increased sitosterol and stigmasterol and increased campesterol signals compared to controls (Compare Fig. 1C–E, see also supplemental Fig. S4). All three plant sterols were readily quantitated in the patients, and the concentrations (Fig. 3A–C) were all above the estimated upper reference range (Table 1). Furthermore, none of the sitosterolemia patients were within the concentration ranges of the control group: the lowest measured concentration of sitosterol and campesterol for the sitosterolemia patients were 70 and 26 μM, respectively. In contrast, the maximal control group concentrations were 17.3 μM (7.8 μM after removal of one outlier) and 16.5 μM, respectively. The median concentrations of sitosterol and campesterol for the sitosterolemia patients were 37- and 15-fold the median control concentrations, respectively (Fig. 3A, B).

In contrast to the 126 controls, all seven sitosterolemia patients had a distinct stigmasterol peak (supplemental Fig. S4C) and were readily quantified (Fig. 3C). The median stigmasterol concentration for the seven sitosterolemia patients was 6.3 μM, and the lowest individual value was 2.8 μM. In contrast, stigmasterol was not above the mLLOQ (1.5 μM) in any of the 126 controls.

The untreated sitosterolemia patient had the highest concentration of both sitosterol and campesterol and second highest stigmasterol concentration (Fig. 3A–C).

### Smith-Lemli-Opitz syndrome: massively increased free 7DHC and likely 8-dehydrocholesterol

Elevated 7DHC is the main biochemical marker for SLOS. In addition, greatly elevated levels of 8-dehydrocholesterol (8DHC) has also been reported, a sterol often poorly or not separated from 7DHC using LC (17, 18).

Control samples generally contained no identifiable 7DHC-peak, and the measured 7DHC concentration in all controls were below mLLOQ (5.7 μM; Fig. 1C and supplemental Fig. S5A). In contrast, SLOS serum produced a massively increased 7DHC peak (Fig. 1F and supplemental Fig. S5B). This 7DHC signal was caused by the combined contribution of 7DHC and an unidentified, greatly elevated compound we suspect is 8DHC (Fig. 1F, magenta arrowhead; see also supplemental Fig. S5C for extended chromatographic separation).

Quantitation of the SLOS 7DHC signal (the combined 7DHC and likely 8DHC) estimated the concentration to 363 μM, a concentration 64-times the maximal upper reference limit for 7DHC (Table 2) and approx. 150-times the concentration estimated by integrating all the peaks in the control-pool serum (supplemental Fig. S5B).

**Table 2:** Free sterol concentrations in SLOS and CTX patient serum

Population	Desmosterol	7DHC	Lathosterol^b^	Cholestanol	Sitosterol
Controls	≤ 0.9	< 5.7	≤ 4.1	≤ 3.9	≤ 6.1
SLOS (n = 1)	0.3	363 ( ≥ 64)^a^^,^^c^	5.5 (1.3)^a^	0.3^d^	1.7
CTX (n = 1)	0.7	24.3 ( ≥ 4.3)^a^	10.5 (2.6)^a^	29 (7.4)^a^	6.8 (1.1)^a^

CTX, cerebrotendinous xanthomatosis; DHC, 7-dehydrocholesterol; ISTD, internal standard; SLOS, Smith-Lemli-Opitz syndrome.

All concentration values are in μM.

a Indicates value above the upper reference limit, and values in parenthesis indicate concentration relative to upper limit (Top row, see also Table 1).

b Lathosterol is semiquantitative and uses the 7DHC-ISTD; recovery and ionization this ISTD may be influenced by the increased 7DHC in SLOS and CTX.

c Measured 7DHC concentration also include minor contribution from presumed 8DHC for the SLOS patient.

d We note that this cholestanol concentration was lower than the lowest cholestanol (0.7 μM) measured in all the 126 control samples.

### CTX: increased free cholestanol, 7DHC, likely 8DHC, and lathosterol

CTX patients fail to produce the bile acid chenodeoxycholic acid and have elevated serum cholestanol. Elevated concentrations of other sterols including 7DHC, 8DHC, lathosterol, and sitosterol have also been reported (19, 20).

CTX serum produced a distinct sterol profile (Fig. 1G and supplemental Fig. S6) compared to controls and the other two diseases: cholestanol (supplemental Fig. S6A) and 7DHC (supplemental Fig. S6B) were both greatly elevated at 29 μM (7.4-times the upper reference limit) and 24 μM (4.3-times the maximal upper limit), respectively (Table 2). A strong signal likely originating from 8DHC was present (Fig. 1G, supplemental Fig. S6B), and lathosterol was moderately increased (Table 2, supplemental Fig. S6C). Sitosterol was slightly above our upper limit.

## Discussion

The reported method measured markedly elevated free sterol concentrations in patients with the rare lipid disorders sitosterolemia, SLOS, or CTX. Many rare diseases are under-researched, and diagnosis can be difficult due to a lack of available diagnostic tools as most clinical sterol measurement methods (except for cholesterol) are laborious and time-intensive.

This project originated from a lack of available biochemical methods in Norway to measure plant sterols in serum. We subsequently developed an LC-MS/MS sterol panel that covered several rare lipid diseases at the same time to increase the diagnostic utility of the method.

LC-MS/MS is today a common tool in clinical chemistry labs (21), and the sterol extraction method reported here is essentially a modified version of a steroid hormone analysis protocol employed daily for clinical routine analysis at OUS (22).

### Free and total sterols concentrations in serum

Concentrations of free sterols in real (nonadulterated) serum are generally expected to be approx. 25–45% of the total (free + esterified) concentration (supplemental Table S1). Our method measured free plant sterol concentrations that were approx. 30–34% of the total concentrations in both controls and sitosterolemia patients (supplemental Fig. S3C, F). For cholestanol, we measured approx. 20% of ERNDIM EQA consensus concentrations for two samples in the normal range (Fig. 2D). In contrast, for the ERNDIM samples that contained 7DHC or cholestanol well above the reference range, our results generally agreed with the consensus concentrations. This is expected as free sterols would be the predominant fraction in these samples due to spiking.

We suggest that for future EQAs, the samples used to compare measured sterol concentrations should preferably be unadulterated samples from affected patients whenever possible and that it is important to report whether a method measured the free or total sterol concentration.

### Simplified biochemical diagnosis of sitosterolemia, a lipid transport disorder

Sitosterolemia is a rare inherited lipid disorder caused by the loss of the sterol transporter *ABCG5/G8* (sterolin). Patients accumulate plant sterols (phytosterols) in their blood and tissues and show a great phenotypic heterogeneity from asymptomatic to skin and tendon xanthomas, premature coronary atherosclerosis, and cardiovascular disease (3, 23). In addition, patients may have increased concentrations of cholesterol, including LDL-C. A substantial portion of patients have hematological abnormalities, including hemolytic anemia and macrothrombocytopenia, and these may present without the “classical” hypercholesterolemia-like symptoms (24). The hematology can be very important: four of our five index patients were initially suspected to have sitosterolemia and referred to our clinic based on abnormal hematology results (the fifth had tendon xanthomas).

Sitosterolemia is often misdiagnosed as familial hypercholesterolemia (FH) and sometimes as an autoimmune hematological disorder (e.g., Immune Thrombocytopenic Purpura) (3, 23, 24). Correctly diagnosing sitosterolemia is important as patients should limit their dietary intake of plant sterols. In contrast, FH patients are usually recommended to do the opposite: increase their intake of margarine or dietary supplements enriched with plant sterols to reduce cholesterol uptake, a potentially harmful diet for patients with sitosterolemia.

Misdiagnosis is mainly due to phenotypic similarity to FH and a lack of available tests specific for plant sterols (25). Traditionally, plant sterol tests measure total (free and esterified) concentrations using laborious sample preparation protocols. The complexity and the resultant high cost of total sterol assays is likely an important reasons for the low availability of clinical plant sterol analyses and a barricade to improved diagnosis of sitosterolemia. We believe the relatively simple and fast method reported herein (Fig. 1A) can improve this: all seven sitosterolemia patients had markedly increased free plant sterol concentrations and could be readily distinguished from 126 controls (Fig 3A–C), consistent with our hypothesis that it should be sufficient to measure the free plant sterol concentration for biochemical diagnosis. Consistent with existing literature (4, 6, 9), we also observed that the proportion of free sitosterol and campesterol was fairly consistent for both sitosterolemia patients and controls over a wide concentration range, with mean free proportions of 34% and 30% for sitosterol and campesterol, respectively (supplemental Fig. S3E, F).

We believe this fairly constant proportion of free sterols in both sitosterolemia and non-sitosterolemia individuals strengthen the underlying rationale for the sufficiency of measuring free plant sterols for the diagnosis of sitosterolemia; however, this should be independently confirmed by others.

Previously considered extremely rare, sitosterolemia has been proposed to be vastly underdiagnosed or misdiagnosed and that the prevalence is closer to 1:200,000 and that as many as 4% of individuals with LDL-C concentrations ≥190 mg/dl may have sitosterolemia (26, 27). The prevalence of sitosterolemia in Norway is currently unknown, but seven known cases in the relevant population of 3.1 million people below 45 years (https://www.ssb.no/en/befolkning/folketall/statistikk/befolkning, accessed 7 May 2024) would indicate a prevalence of approx. 1:440,000 or higher.

### SLOS—a congenital malformation and mental retardation syndrome

SLOS is a multiple congenital malformation and mental retardation syndrome caused by a deficiency of 7DHC reductase, an enzyme important for the biosynthesis of cholesterol. The clinical incidence of SLOS is estimated to 1:10,000 to 1:70,000, and patients have a wide phenotypic spectrum (28, 29). Patients often have low or low-normal cholesterol concentration, and the concentration of 7DHC and the isomer 8DHC are typically increased more than 50-fold (17, 30). Currently there is no cure for SLOS (31).

Our method clearly separated the SLOS patient from the controls. In normal serum, 7DHC was low (<5.7 μM) and generally did not produce a visually distinct signal (supplemental Fig. S5A). In contrast, the SLOS serum produced a vastly increased 7DHC signal that dwarfed all the other measured sterols (Fig. 1F). The estimated 7DHC (including the presumed 8DHC) was estimated to be more than 100-fold increased compared to typical normal serum concentrations (supplemental Fig. S5B) and ≥ 64-times our upper reference limit (Table 2).

We note that due to a lack of baseline separation of 7DHC from other sterols, the method had a relatively high mLLOQ (supplemental Table S6) and a resultant high (maximal) upper reference limit for 7DHC (Table 1). There are reported cases of SLOS patients with very minor elevations of 7DHC that would likely be below our mLLOQ (18, 32). Because spiking the control serum with 2 μM 7DHC produced a strong qualitative signal for 7DHC (supplemental Fig. S5A), we suggest that the 7DHC-extracted ion chromatogram should always be manually inspected for signs of elevated 7DHC concentrations.

### CTX—a severe but treatable disease

CTX is a rare but treatable disease caused by a defect in sterol 27-hydroxylase (*CYP27A1*), an enzyme involved in bile acid production. Patients have low or normal cholesterol and typically 4- to 5-fold increased serum concentrations of cholestanol (a stanol). Cholestanol can accumulate in several tissues including tendons, the eye-lens, and the central nervous system, and the severity of the disease ranges from mild symptoms to severe irreversible and progressive degeneration, including cognitive impairment. Progression of the disease can be prevented or stabilized using bile acid replacement therapy (e.g., chenodeoxycholic acid), and this lowers plasma cholestanol levels. However, full treatment effect generally requires early diagnosis, and this highlights the importance of access to diagnostic analyses. (1, 33, 34). In addition to cholestanol, 7DHC, 8DHC, lathosterol, and sitosterol have been reported to be elevated in CTX (19, 20). Recently, cholestanetetrol glucuronide has emerged as promising marker for CTX screening (35).

Measured cholestanol concentration for the CTX patient (29 μM; Table 2) was 7.4-times the concentration of our upper reference limit (3.9 μM), and 3.4-times the maximal concentration measured for a control group individual (deemed to be an outlier; see distribution in supplemental Fig. S2). 7DHC was elevated in our patient, and we observed an increased peak that was likely caused by elevated 8DHC. Lathosterol was also increased (10.5 μM; upper reference 4.1 μM), and we observed that all the campesterol transitions contained two elevated and unidentified peaks that were not present in other samples (supplemental Fig. S6D).

Although we only had access to serum from one SLOS and one CTX patient, we believe our method is a valuable tool for diagnosis of SLOS and CTX in general: most SLOS patients have greatly elevated 7DHC concentrations compared to controls (17, 30). Similarly, a recent large study that reported the mean cholestanol concentrations for 43 CTX patients (untreated) was 6-times higher than the maximum normal concentration, and the patient with the lowest cholestanol concentration was almost twice the normal upper range (36).

### Desmosterolosis and lathosterolosis

Desmosterolosis and lathosterolosis are two extremely rare, autosomal syndromes. Patients typically have multiple congenital anomalies and a partly overlapping phenotype to SLOS (29). Plasma concentrations of desmosterol were reported to be more than 100-fold increased in desmosterolosis compared to controls (37, 38). Similarly, lathosterolosis patients have greatly elevated serum lathosterol concentrations (39, 40). Recently, a very mild case of lathosterolosis was reported, but this patient also had greatly elevated lathosterol concentrations (41).

We did not have access to serum from desmosterolosis or lathosterolosis patients. We can therefore not confirm that our method measures increased concentrations in these patients. However, our results showed that the method is capable of measuring increased, free concentrations of the two sterols in serum (e.g., supplemental Fig. S1; supplemental Tables S6–S8). Because desmosterol and lathosterol are both present in free form at a significant fraction in plasma (5), we assume that desmosterolosis and lathosterolosis patients also have greatly increased free sterol concentrations; however, this should be confirmed in future studies.

### Extrapolation to estimate concentrations in pathological samples

The prepared calibrators covered normal serum concentrations but had a limited linear range (supplemental Table S6). However, pathological concentrations in undiluted serum could be estimated by calibrator-extrapolation with a bias that was not clinically important for diagnosis (supplemental Table S6).

In summary, the reported method allows for simple and fast analysis of the free concentration of seven diagnostic sterols elevated in rare lipid disorders. Seven sitosterolemia, one SLOS, and one CTX patient were distinguished from controls, indicating that the free concentration is sufficient for biochemical diagnosis of sitosterolemia and likely SLOS and CTX. However, we do not claim that our method is better than total concentration methods. Our rough estimate of the cost (consumables and materials) of the analysis is $15 (US Dollar) per sample.

A limitation of this study is the small number of affected patients, an inherent obstacle in studying rare disorders. In addition, the control sample population was predominantly composed of adults. Future research should address this, include demostolosis and lathosterolosis patients, and preferably include mild, moderate, and severe cases. Especially SLOS patients with very minor elevations of 7DHC may be missed by the reported method as their concentration may be below our mLLOQ. We therefore suggest that future studies should also investigate variations in the chromatography to better resolve 7DHC from interferences. It is also preferable to include additional patient groups (e.g., infants and patients with liver disease) to investigate how the method performs in a larger clinical setting, including calculation of test sensitivity and specificity.

## Data availability

The data that support the findings of this study are not openly available due to reasons of sensitivity, but are available from the corresponding author (Alexander Bauer Westbye, alwest@ous-hf.no) upon reasonable request. Data are located in controlled access data storage at Oslo University Hospital.

## Supplemental data

This article contains supplemental data.

## Conflict of interest

The authors declare the following financial interests/personal relationships which may be considered as potential competing interests: P. M. T.: Honoraria from Sanofi for lectures. E. E. A.: Honoraria from Amarin, Novartis and Sanofi for lectures. K. R.: Honoraria from Amgen, Amarin, NovoNordic, Novartis, Sanofi for lectures. A. B. W., S. R. D., and P. M. T.: Financial support from The South-Eastern Norway Regional Health Authority to the Hormone Laboratory (∼10,000€). The not-for-profit foundation “Stiftelsen Nils Normans forskningsfond til Hormonlaboratoriets fremme” has supported various research and development work at the Hormone Laboratory, including the development of submitted method. E. A. A., L. L. D., and K. R.: Financial support from The South-Eastern Norway Regional Health Authority to the Lipid Clinic (∼40,000€). Y. T. B., A. L. S., and U. D. declare no competing interest.
