# Supplementary material for: A sterol panel for rare lipid disorders: sitosterolemia, cerebrotendinous xanthomatosis, and Smith-Lemli-Opitz syndrome
Source: J Lipid Res. 2024 Nov 19;66(1):100698. doi: 10.1016/j.jlr.2024.100698 (PMC11714705; doi:10.1016/j.jlr.2024.100698)
Supplement: Supplemental data [file mmc1.docx]

# **Westbye et al. A Sterol Panel for Multiple Rare Lipid Disorders: Sitosterolemia, Cerebrotendinous Xanthomatosis and Smith-Lemli-Opitz Syndrome**

# **Supplemental material**

## **Detailed Materials and Methods**

### **Patient samples**

Serum from participants in the on-going study “Sitosterolemia in Norway”, including seven sitosterolemia patients (previously verified by genetic tests) from five unrelated families referred to the Lipid Clinic (Oslo University Hospital; abbreviated OUS), were collected into silica-gel tubes (Vacuette Serum Gel 5 ml, G456073R), carefully mixed and centrifuged after 30-120 minutes (2100 g, 21 °C, 15 minutes). Serum samples were aliquoted (1.2 mL; Sarstedt Micro Tubes, 2mL, Polypropylene) and stored at −80 °C until analysis.

Serum from one SLOS and one CTX patient (previously verified by genetic tests; SLOS was also verified biochemically) originated from left-over serum samples stored at -20º C at the Norwegian National Unit for Diagnostics of Congenital Metabolic Disorders (OUS).

The control samples from presumed healthy (in respect to rare sterol disorders) individuals were collected from left-over, anonymized patient serum samples received as part of routine diagnostic testing at the Hormone Laboratory (OUS). The samples, received in a variety of collection tubes and stored -20 °C for less than 1 month, were haphazardly selected from routine tests of the biomarkers free T4 (n=40; thyroid disease marker; no fasting requirement), C-Peptide (the connecting peptide of proinsulin; n=42; diabetes marker; usually fasting) and Carboxy-terminal cross-linked telopeptide of type 1 collagen (n=44; osteoporosis monitoring; sample to be taken fasting). The control-serum pool was obtained by combining approx. equal volumes from 20 samples originating from osteoporosis and diabetes routine samples.

### **Sterol standards, solvents and calibrator preparation**

Regular and deuterated sterol standards (listed in Table S2), lipoprotein deficient serum from human plasma (LD-serum; Sigma-Aldrich, Cat. S5519), purified water and organic solvents (LC-MS or analytical grade) were acquired from Merck (Merck AB Norway).

Sterols not supplied in solution were initially weighed out using a microgram scale and dissolved in dichloromethane (DCM) at high concentrations (~1 mg/mL). Individual 100 µM stocks of each sterol or ISTD were prepared in isopropanol (IPA) by volumetric dispensing of DCM-stocks, evaporation under N_2_ and reconstitution in IPA. All stocks were stored in amber glass vials with Teflon-septum screw caps (“HPLC-vials”) at -20 °C.

Accurate volumetric transfer of volatile solvents (e.g. DCM) was performed using positive displacement pipettes (Microman E, Gilson). Multipette E3 (Eppenford) positive displacement pipettes were used for repetitive volumetric dispensing (e.g. ISTD-addition). We note that 7DHC is prone to oxidation, and exposure to oxygen and UV-irradiance should be limited (1, 2).

### **Analytical method development**

#### Optimization of LC-MS/MS parameters

Source and fragmentation parameters were optimized individually for each sterol by manual infusion using relatively high concentrations (approx. 20 µM) and electrospray ionization in positive mode (ESI+) using a Sciex QTRAP 6500+ MS instrument equipped with a IonDrive Turbo V ion source. Precursors ions were identified based on intensity and an observed trend of water-loss (i.e. [M+H-H_2_O]^+^ ion-formation). Up to eight product ions were initially selected based on MS^2^-scan performed with a ramping of collision energy. The transitions were manually optimized for declustering potential (DP) and cell exit potential (CXP), and investigated for suitability in preliminary LC-MS/MS methods using multiple reaction monitoring (MRM). The chosen transitions, compound specific- and source parameters for all the sterols are listed in Tables S3 and S4. Quantitation was performed using deuterated-equivalents as ISTD (isotope dilution), except for lathosterol that was semi-quantitated using the 7DHC ISTD; cholesterol was measured but not quantitated.

Several chromatographic conditions were investigated using both standards and purified serum before deciding on the final method: five µL sample was injected on a phenyl-hexyl column (Phenomenex Kinetex 1.7 µm 100 Å, 100 x 2.1 mm; Cat no. 00D-4500-AN). The mobile phases consisted of H_2_O 0.2% formic acid (Phase A) and acetonitrile (ACN) 0.2% formic acid (Phase B). The LC method started at 72.6 % B and was linearly ramped to 76.4 % B over 6.4 minutes, then quickly ramped (0.1 min) to 100 % B to flush column for 1.5 min. The method used a one minute re-equilibration and the flow rate was 0.5 mL/min. After completion of an analysis batch, the system was flushed extensively with 100% IPA.

Verification of chromatographic separation of free sterols from steryl-esters was performed using atmospheric-pressure chemical ionization (APCI), previously reported to cause in-source fragmentation of steryl-esters into the corresponding sterol’s [M+H-H_2_O]^+^ ion (3) and the following modification to the LC-MS/MS method: time 0 to 6.4 min was unaltered. From 6.4 to 8.0 min the gradient was linearly ramped to 100 % B, and 100 % B was kept for 7 min. The MS-acquisition was not-scheduled and limited to cholesterol, sitosterol and campesterol MRMs (dwell time 80 ms). For the additional qualitative separation of 7DHC from the unidentified isomer in the SLOS-serum (and lathosterol from cholesterol), an 18 min gradient separation from 70-76 % B was used.

#### Optimization of sample extraction

Optimization of sample extraction focused on the recovery (measured peak area) of sitosterol, campesterol and cholestanol from serum pool using 96-well format supported liquid extraction plates (Isolute SLE+, 400 µL, Biotage. Cat no. 820-0400-P01). Different sample volumes and the organic extraction solvents (ethyl acetate (EtOAc), hexane (Hex), DCM and mixtures thereof) were investigated. Pretreatment by addition of IPA (1:1 IPA:serum) or IPA addition followed by sonication (10 min) in a ultrasonic bath was also investigated.

#### Sample equilibration time and potential esterification by LCAT

Lecithin:cholesterol acyltransferase (LCAT) activity present in serum could hypothetically influence quantitation by esterifying the ISTDs during the equilibration-phase of sample preparation. This was investigated by incubating serum-pool (diluted as for sample preparation) with 5 µM deuterated sterols. Samples were incubated at RT (22 °C) or 37 °C for 15, 30 or 60 min then transferred onto ice. Results were compared to samples stored on ice for 60 min and with samples transferred directly to SLE without equilibration.

#### Final sample extraction procedure

Calibrators were matched as closely as possible to serum matrix using LD-serum. 2-times (2x) concentrated calibrator-stocks (Table S5) and 20 µM ISTD-stock were prepared in IPA and stored in amber HPLC-vials (2 mL; Agilent, Cat no. 5182-0716) with teflon-septum screw caps (Agilent, Cat no. 5182-0717) at -20 °C. For each sample preparation, a working stock of ISTDs (0.8 µM (final) in 4:6 vol:vol IPA:H_2_O) was prepared fresh.

Samples and calibrators were prepared in 96-well plates (Strata 2 mL Conical Polypropylene; Phenomenex, Cat no. AH0-7194) by combining (in order): 300 µL dH_2_O, 50 µL serum or LD-serum, 25 µL 2x calibrator-stocks or IPA and 125 µL of ISTD-working stock. Plates were sealed with adhesive film, and incubated at 600 rpm (ThermoMixer C, Eppendorf) at RT (22 °C) for 30 min. 400 µL was transferred to an SLE-plate and allowed to incubate for 5 min. Sterols were gravity-eluted into collection plates (Strata 2mL) by applying 500 µL EtOAc twice with 5 min incubation after each addition. Solvent was evaporated under N_2_ flow at 55 °C and the samples resuspended in 500 µL of 70% ACN in dH_2_O (vol/vol) and analyzed same day.

Sitosterolemia, SLOS and CTX serum was diluted in LD-serum to accurately quantitate analytes present at concentrations above the mULOQ. Note that 7DHC is prone to oxidation (1, 2).

### **Method validation**

#### Quantitation limits and calibrator linearity

The lower limit of quantitation (LLOQ) in LD-serum was investigated for concentrations down to 0.05 µM (7DHC, cholestanol, stigmasterol, campesterol and sitosterol), 0.025 µM (desmosterol) or 0.0125 µM (lathosterol). The LLOQ was established based on the presence of a visually identifiable peak, a signal to noise above 10 for all six replicates, and an intraday CV below 15% using LD-serum calibrators. Because of the increased complexity of real serum compared to LD-serum, the method lower limit of quantitation (mLLOQ) for each sterol was manually adjusted (increased) from the LLOQ based on the following criteria: The mLLOQ was pragmatically increased for the analytes stigmasterol, desmosterol and 7DHC to allow visual peak identification, reliable integration and ion ratio acceptance (< 20% deviation acceptance) in real human serum based on manual inspection of analyzed serum pool, individual serum samples and spiking experiments. For the other sterols, mLLOQ was increased from the LLOQ to cover the expected lower concentration in normal samples based on analysis of serum pools and individual samples.

The linear range of the calibrators was investigated using linear regression (1/x^2^ weighting). Plant sterols exhibited a marked deviation from linearity at high concentrations, and the method upper level of quantitation (mULOQ) was established as the most concentrated calibrator that had an accuracy > 85 % using the linear calibration model.

We did not attempt to determine the lower limit of detection in accordance with the current Clinical and Laboratory Standards Institute (CLSI) C62 guideline (2022; Section 6.6.3.1).

#### Imprecision, accuracy, matrix effects, absolute recovery and carryover

Intraday (same day) percentage coefficient of variation (%CV) was calculated from ten replicates, except for sitosterolemic and SLOS serum. Inter-day (between days) %CV was calculated by the “mean-of-means” method: six replicates were prepared and analyzed on four days and the mean for each day was calculated. A grand mean and %CV was then calculated from the four means. Due to limited sitosterolemia pool- and SLOS serum, intraday %CV was calculated from six (diluted) or three (undiluted) and twelve (diluted) or three (diluted) replicates, respectively.

Method accuracy was investigated by comparing measured increase after spiking (“spike and recover”) and by analyzing samples from the “European Research Network for evaluation and improvement of screening, Diagnosis and treatment of Inherited disorders of Metabolism” (ERNDIM): Accuracy of measured increase after spiking was calculated from the percentage difference in measured concentrations of serum pool spiked with known concentrations ($C_{Spiked}$) of sterols before sample extraction ($C_{Meas, Spike}$) and non-spiked controls ($C_{Meas, 0}$) using $\frac{C_{Meas, Spike}-C_{Meas, 0}}{C_{Spiked}}$ . Spiking was performed at three levels (addition of 2, 5 or 10 µM). For stigmasterol and 7DHC, accuracy was calculated using the difference to serum spiked with 2 µM.

Accuracy for 7DHC and cholestanol was further investigated using two ERNDIM controls (“L1” and “L2”) included with the Internal Quality Control System (IQCS) Special Assays in Serum and eight previously received ERNDIM external quality assurance (EQA) samples. For the EQA samples, measured concentrations were compared to the ERNDIM consensus concentrations as follows to not disclose the confidential consensus concentrations: Accuracy was calculated by diving our result with the ERNDIM-reported median concentration. A Z-score (i.e. the number of standard deviations from the mean) was calculated by subtracting the ERNDIM-reported mean and dividing this by the ERNDIM standard deviation for the consensus mean.

Matrix effects on signal intensity were investigated by comparing post-extraction spiked samples to standards or ISTDs in pure solvent: six replicates of LD-serum or serum-pool were purified without the addition of internal standards. The samples were reconstituted in 500 µL of 70% ACN containing 0.16 µM (2 µM relative to serum volume) of sterols (LD-serum) or ISTD (serum-pool). Matrix effects were calculated from the peak areas (A) using $\frac{A_{Matrix}-A_{Solvent}}{A_{Solvent}}$.

The effect of sample dilution and the feasibility of extrapolation above mULOQ using undiluted sitosterolemic or SLOS serum was investigated by diluting serum with LD-serum prior to sample extraction. The difference in the calculated concentrations obtained for the undiluted serum by linear extrapolation of the calibration curves was compared to the concentration obtained for the diluted samples.

Absolute recovery (i.e. the percentage of the sterols in serum recovered by sample extraction) experiments were performed by adding equivalent concentration of ISTD to serum pool samples spiked with zero or 10 µM of all analytes (except cholesterol) before or after sample preparation. Absolute recovery was calculated by dividing the peak area ratio of ISTD to analyte of samples spiked before and after.

Carryover effects were investigated by injecting solvent-blanks after normal and high-concentration samples. No indication of carry-over contamination was apparent by manual inspection of blank injections after the analysis of normal serum or undiluted sitosterolemia or SLOS-serum.

### Plant sterol analysis by Karolinska Institutet

11 serum samples from participants in “Sitosterolemia in Norway”, selected to cover a wide concentration range of free sitosterol (0.4 to 144 µM) and campesterol (0.7 to 97.3 µM), and a serum pool were sent to Karolinska Institutet (Stockholm, Sweden) for analysis. In brief, free sterols in serum was measured by diluting sample in water, adding d6-sitosterol (ISTD), EtOH and cyclohexane. For total sterols, an additional saponification step with NaOH for 1-2h at 65 °C with shaking was performed. Sterols were purified by liquid-liquid extraction, evaporated, derivatized to trimethylsilyl ethers at 60 °C for 30 min, evaporated then reconstituted in hexane and analyzed by GC-MS (4). Deuterium labelled sitosterol (d6) was used as the ISTD for both sterols. Measured concentrations values (in µg/mL) were converted to molar equivalents using 414.71 (sitosterol) 400.68 g/mol (campesterol).

### Estimated upper reference limit for clinical guidance

Upper reference values for clinical guidance was estimated from the control group (126 samples). For desmosterol, cholestanol and sitosterol, one value inferred to be an extreme outlier (possibly a patient with a liver disease) was removed after manual investigation. Values below the quantitation limits (one desmosterol, two lathosterol values) were imputed as ½ the quantitation limits and one value above the quantitation limit (lathosterol) was calculated by extrapolating the calibration curve. The upper reference limit of a 95 % one-sided reference interval was estimated by calculating the 95^th^ percentile, and a 90% confidence interval (CI) for the limit was calculated by bootstrapping (10 000 iterations) for each analyte. For stigmasterol and 7DHC, the upper limit was set to be below mLLOQ (i.e. unknown, but less than mLLOQ).

### Software and data analysis

The LC-MS/MS was controlled by Sciex Analyst (v 1.7.3) and quantitation performed using Sciex OS Analytics (v 3.0.0.3339) with Low smoothing, and all peaks were manually inspected. Data handling was performed using Microsoft Excel (2016) and R (v 4.3.0) using RStudio (v 2023.09.01) (5, 6). Statistical summary parameters for the control population, upper reference limits and statistical tests were performed in R. The upper limit was estimated as the 95^th^ quantile for the population calculated using *quantile(probs = 95/100).* The 90 % CI of the limit was found by calculating the 5^th^ and 95^th^ percentiles of upper limits generated by bootstrapping (i.e. from 10,000 calculated upper limits (95^th^ percentiles) generated after randomly selecting concentrations with replacement using *sample(replacement = TRUE*). Passing-Bablok regression of plant sterol concentrations was performed using the R package *mcr* with setting *method.reg = “PaBa”*. Statistical significance of free plant sterol concentrations in sitosterolemia-patients to controls was tested using the non-parametric Mann-Whitney U test using *coin::wilcox_test(distribution = “exact”)* (7). The “Tidyverse”, including ggplot2, was used for general data wrangling, Bland-Altman plots and general visualization (8).

## **Supplemental results**

### **Development of an easily implementable sample extraction and LC-MS/MS method for measurement of free sterols in human serum**

Method development (sample extraction and LC-MS/MS analysis) focused on developing a simple sample extraction method suitable for clinical laboratories that did not use base-hydrolysis (saponification) or liquid-liquid extraction and that minimized the use of chlorinated solvents (often used in sterol analyses). The LC-MS/MS method development similarly focused on maintaining simplicity and having a sufficiently short run time for the analysis of a moderate number of samples from large hospitals and/or larger screening projects.

All sterols formed precursor ions corresponding to [M+H-H_2_O]^+^ (a net loss of 17 mass units) using ESI. Sterol standards separated well on a phenyl-hexyl column using both MeOH and ACN, however ACN proved essential to achieve sufficient chromatographic separation from interferences in serum samples (not shown). The two stereoisomers desmosterol and 7DHC were well separated from each other, while lathosterol and the stereoisomer cholesterol were only partially separated (see Fig S6C). However, the transition 369.3 to 301.2 m/z was much more specific for lathosterol than cholesterol (compare lathosterol and cholesterol-XIC in Fig 1B and 1C), and this was used as the main transition (“Quantifier”) to monitor and semi-quantitate lathosterol. We furthermore verified that the method separated sterols from steryl-esters; the latter were retained much more strongly on the phenyl-hexyl column (Fig S7).

Sample extraction optimization mainly focused on plant sterols and cholestanol: ethyl acetate (EtOAc) and dichloromethane (DCM), either 100% or mixed with hexane, were both suitable for sample extraction (Fig S8A and S8B). Treatment with IPA or IPA and sonication prior to extraction reduced plant sterol recovery and was not investigated further (Fig S8A). EtOAc was favored over DCM as it is a less hazardous solvent (not chlorinated) and more suitable for routine-laboratory use. Addition of hexane to EtOAc did not offer substantial improvements (Fig S8B). Smaller sample volumes produced substantially increased plant sterol signal in the extract compared to larger volumes (Fig S8C). In contrast, desmosterol and cholestanol were less affected by volume, and we settled on standardizing the serum volume to 50 µL. Incubation at different temperatures did not markedly alter quantitation of the plant sterols, indicating that there was negligible esterification of the ISTDs (by serum LCAT-activity (9)) during sample preparation (Fig S8D).

### **Validation and method suitability**

Desmosterol, cholestanol, campesterol and sitosterol were baseline-separated from other compounds and quantifiable in control group serum (Fig 1B and 1C) and allowed for reproducible and accurate integration (Table S7 and Table S8). 7DHC, lathosterol and stigmasterol were either not present in control serum and/or fully separated from interferences (Fig 1C, see also Figs S4C, S5A and S6C):

Stigmasterol was generally present at too low concentrations in the control group to quantitate using our method: it formed a barely detectable peak next to a closely migrating interference in most samples (Fig S4C). Spiking control serum with 2 or 10 µM (Fig 1D) produced a markedly elevated peak. Quantitation of the increase (2 µM compared to 10 µM) was possible with an accuracy of 129% and spiked control serum had low imprecision (Table S7 and Table S8). Stigmasterol was markedly elevated in the sitosterolemia patients (Figs 1E and S4C).

7DHC was not baseline-separated from interferences in control serum (similar “multi peak” profiles were present in four m/z-transitions investigated; Fig S5A). However, serum spiked with 2 µM or more 7DHC produced visually distinct 7DHC peaks at a RT corresponding to a shoulder and near a local low point (valley) in the 7DHC-“multi peak-profile” (Fig S5A). Quantitation of the increase from 2 to 10 µM was possible with good accuracy and intraday %CV (Fig S1, Table S8). However, acceptable inter-day imprecision was only obtained after spiking with 4 µM (resulting peak integrated to 5.7 µM, Table S7). Because 2 µM spiked control serum produced a large and distinct 7DHC peak (Fig S5A), we expect that the “true” upper reference limit for 7DHC was likely well below the mLLOQ of 5.7 µM.

Both a SLOS and a CTX patient had markedly elevated 7DHC signal (Figs 1F, 1G, S5B and S6B). Closer examination of the 7DHC peak in SLOS serum indicated that it was composed of the signal from two compounds. Using an extended chromatographic method, we confirmed that the SLOS 7DHC peak formed using the regular chromatographic method had a minor contribution from another compound (Fig S5C). We assume that this later-migrating peak is the stereoisomer 8DHC that has previously been reported to be elevated in SLOS, and elevated in at least some CTX patients, and to be difficult to separate from 7DHC using chromatography (10, 11).

Separation of lathosterol from the steroisomer cholesterol in serum using LC-MS/MS is reported to be difficult, due to poor chromatographic separation and because of the very high cholesterol concentrations relative to lathosterol (12, 13). Our method partially separated lathosterol from cholesterol in serum using the transition 369.3 to 301.2 m/z that we found was much more specific for lathosterol than cholesterol. The peak was visually increased after spiking (Compare Fig 1C and 1D) and quantitation using the 7DHC ISTD indicated that our method could semi-quantitate increased lathosterol concentrations (Fig S1) with an accuracy between 64 and 76% (Table S8). Close-to baseline separation from cholesterol was possible using the extended method (not shown). Similar to previous reports (10), we observed that serum from a CTX patient contained markedly elevated lathosterol compared to controls (Fig S6C).

## **Supplemental Discussion**

### **Considerations for clinical laboratories**

Total plant sterol tests are laborious and require saponification (base-hydrolysis), often repeated liquid-liquid extractions and derivatization. In contrast, the reported method is simple and fast and should be relatively easy to implement in clinical laboratories accustomed to LC-MS/MS (e.g. endocrinology or pharmacology labs). Extraction is performed with ethyl acetate using SLE in a 96-well format, but could also be performed using single-sample extraction columns (e.g. Biotage Isolute SLE+ 400 µL columns; Biotage part no. 820-0055-B; not tested). Extraction and preparation for LC-MS/MS analysis of one batch of up to 80 samples takes approx. four hours for a skilled technician (including incubation, evaporation and reconstitution). The relatively rapid LC-MS/MS analysis time (<10 min) allows a full 96-well plate to be analyzed over-night (approx. 16h). We believe this also makes the analysis suitable to screen patient-populations with clinical symptoms of FH but no established genetic cause to uncover potential un- or misdiagnosed sitosterolemia-cases.

We caution that our method only semi-quantitates lathosterol, but we expect that the current method would clearly distinguish even mild lathosterol cases from controls. The limitations to accurately quantitate lathosterol is due to incomplete separation from cholesterol (a stereoisomer of lathosterol) that is measured at a low efficiency using the “lathosterol-specific” transition 369.3 to 301.2 m/z (see Fig S6C), making accurate and reproducible integration of lathosterol difficult. Furthermore, the ISTD used for lathosterol (the 7DHC ISTD) is not expected to fully compensate for ion-suppression – however this latter limitation can be readily overcome by incorporating an isotopically-labelled lathosterol ISTD.

7DHC is known to be readily oxidized by radical chain reactions (1, 2). The 7DHC ISTD will correct for oxidation during sample preparation, but not correct potential degradation occurring prior to addition of the ISTD. Except for shielding samples from direct daylight (UV irradiation) and storing samples frozen, we did not take additional measures to reduce 7DHC oxidation. Some authors advocate to use the protectants butylated hydroxytoluene and triphenylphosphine when measuring 7DHC (1), however we have not investigated if these offer benefits to our method. Because measured 7DHC concentrations in the ERNDIM samples agreed well with expected values (Fig 2C and Table S9), we believe oxidation is not a particular concern using the reported method.

## **Supplemental Figures**


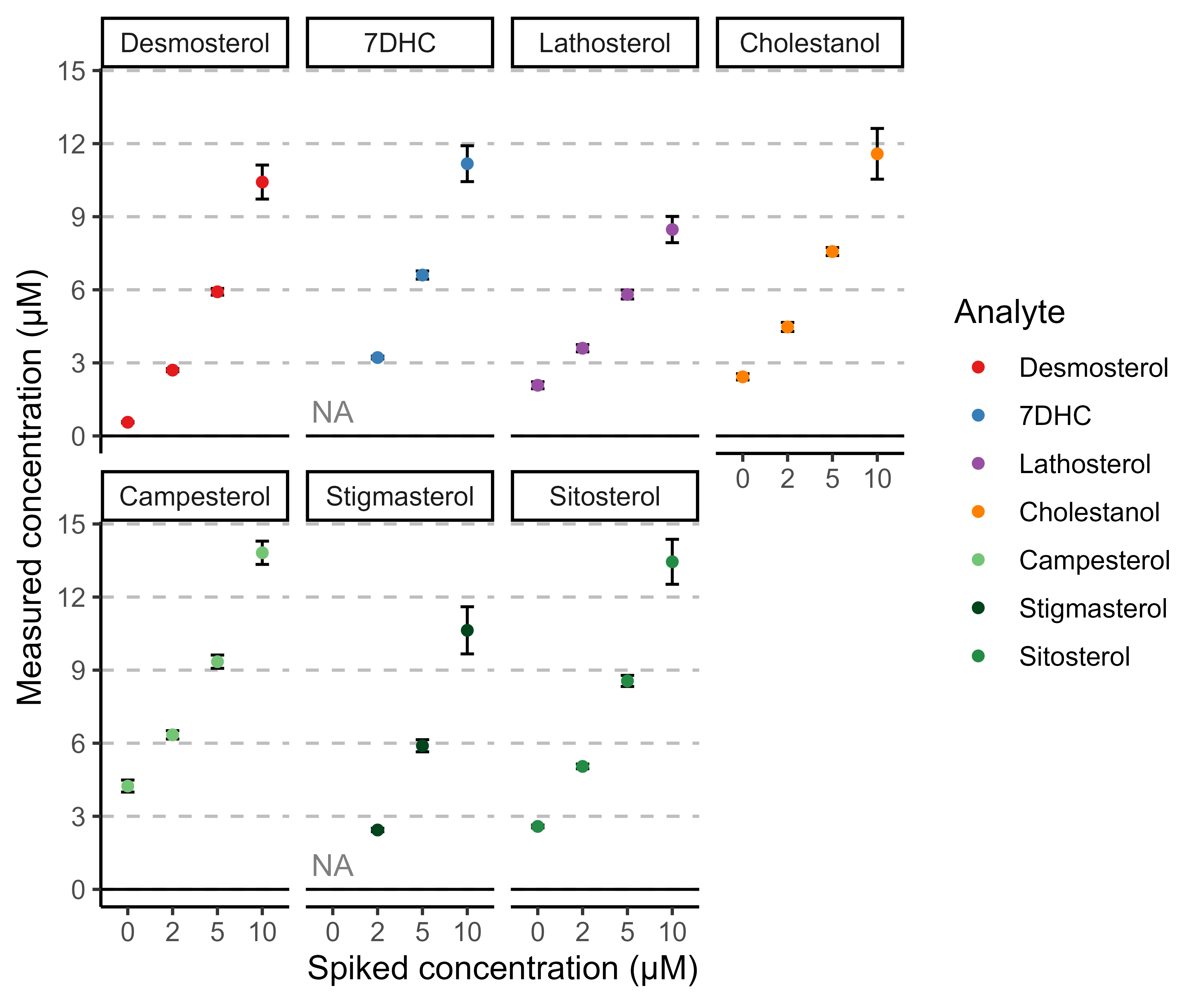


**Fig S1 Method response to added sterols (“spiking”)**Response of method to sterols added (“spiked”) to control serum. For 7DHC and stigmasterol the concentration was below mLLOQ in non-spiked samples. Note reduced response to lathosterol.





**Fig S2. Free sterol concentrations in the control group**
Measured concentrations of free sterols and cholestanol in serum from 126 presumed non-affected/healthy (in respect to rare sterol disorders) patients. Concentrations for each subgroup (original analysis requisition; **A**) and combined into one group (**B**).
C-peptide: Connecting peptide (of proinsulin). C-term telopep I col: C-terminal telopeptide of type I collagen. Boxplot whiskers extend from first or third quartile to the smallest or largest value, respectively, no further than 1.5-times the inter-quartile range.





**Fig S3.** **Method comparisons**Comparison of measured sitosterol (**A**, **C** and **E**), campesterol (**B**, **D** and **F**), 7DHC (**G**) and cholestanol (**H**) concentrations. **A** and **B**. Bland-Altman plots of concentrations measured by reported method to free plant sterol concentrations measured by Karolinska Institutet (KI). **C** and **D**. Passing-Bablok comparison of concentrations measured by reported method to total plant sterol concentrations (KI). **E** and **F**. Calculated proportion of free sterols (KI) to total concentrations (KI), arranged by total concentration. **G** and **H**. Calculated Z-scores from concentrations measured by reported method to reported consensus concentrations (mean) and standard deviations of ERNDIM external quality assurance samples, arranged based on the consensus concentration.





**Fig S4. Elevated plant sterols in sitosterolemia patients**
Plant sterol signals (quantitative transition) for sitosterol (**A**), campesterol (**B**) and stigmasterol (**C**) in control serum (pool) and a representative sitosterolemia patient. Note relative intensity to ISTDs, and the absence of a stigmasterol peak in control, but intense peak in sitosterolemia. Y- and X-axes show intensity (arbitrary units) and time (minutes), respectively.





**Fig S5. Elevated 7DHC and presumed 8DHC in a Smith-Lemli-Opitz Syndrome patient.**The 7DHC signal (quantifier transition) and calculated concentrations for control serum (pool), spiked controls and Smith-Lemli-Opitz Syndrome (SLOS) serum. Representative peaks using the reported (“regular”) LC-MS/MS method (**A**, **B**) and using the extended chromatographic separation (**C**). **A.** Control serum contained very low 7DHC levels, but a marked peak is visible when serum is spiked with 2 µM or more 7DHC. Note that the retention time (RT) of 7DHC is between the RTs of the two peak-maxima present in control serum (the left-most peak is from the steroisomer desmosterol, also spiked). **B.** SLOS serum contained massively increased 7DHC concentrations compared to control. The calculated concentrations below the peaks are corrected for dilution, and show concentrations calculated by integrating all the peaks as indicated. For undiluted and 10-fold diluted SLOS serum, the calibration curve was extrapolated (from the mULOQ 20 to 242 and 35.3 µM, respectively). Note the ISTD peak height. **C.** Separation of 7DHC from a closely-migrating and elevated analyte in SLOS serum presumed to be 8DHC using the extended chromatographic method.
Y- and X-axes show intensity (arbitrary units) and time (minutes), respectively.





**Fig S6 Elevated sterols and cholestanol in a Cerebrotendinous Xanthomatosis patient.**Qualitative difference in the XIC (quantifier transition) for cholestanol (**A**), 7DHC (**B**), lathosterol (**C**) and campesterol (**D**) for control serum (pool) compared to serum from a Cerebrotendinous Xanthomatosis (CTX) patient. Note the relative intensity of the lathosterol-peak compared to the ISTD and the peak originating from cholesterol (a stereoisomer of lathosterol). For **D**, note the presence of elevated unidentified peaks in the campesterol-transition.


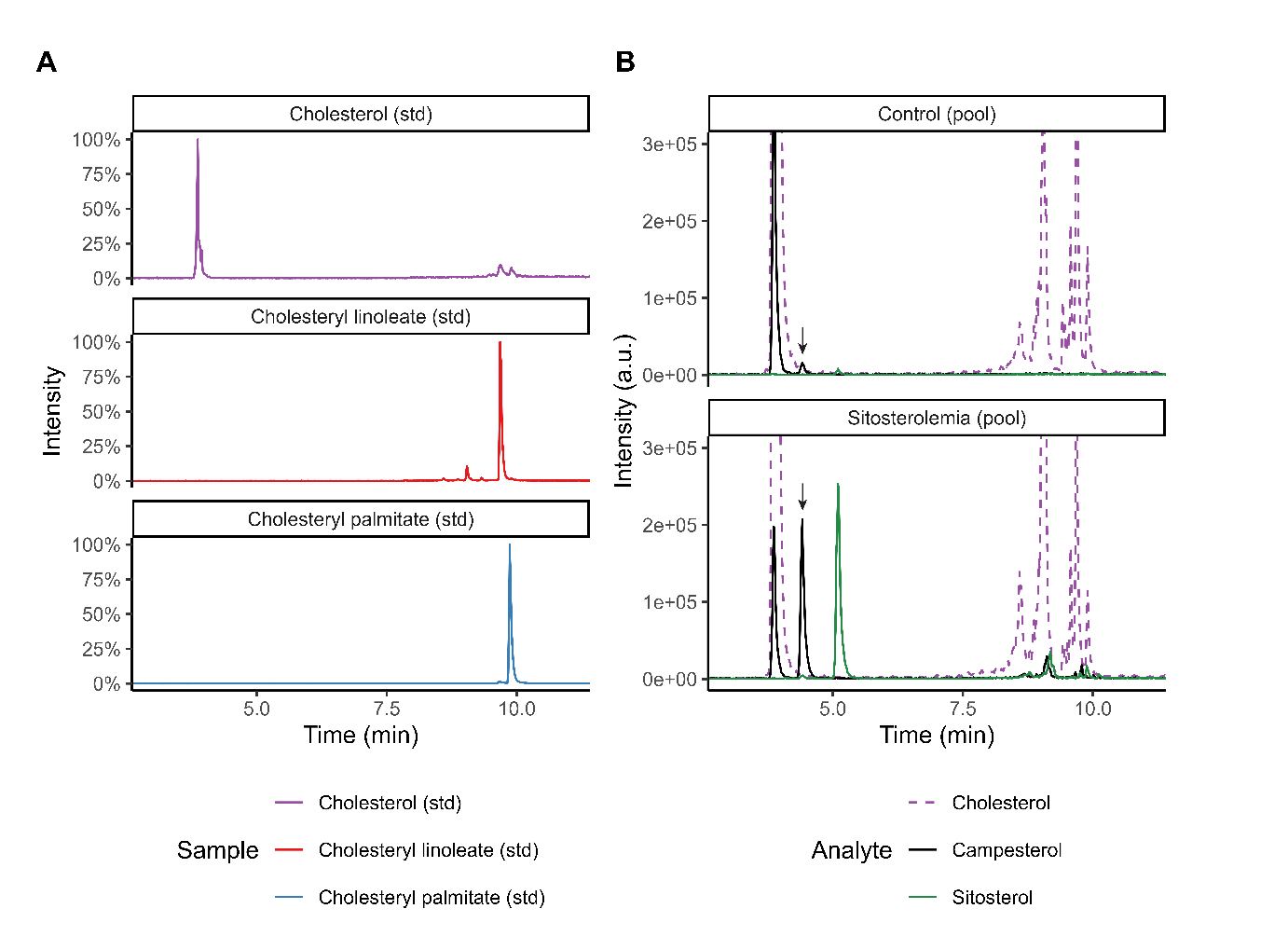


**Fig S7. Chromatographic separation of free sterols from steryl-esters**
Chromatographic separation of sterols from steryl esters for pure standards (**A**) or in serum pools (**B**). **A.** Chromatographic retention of cholesterol and the corresponding linoleate (C18, two double bonds) or palmitate (C16, saturated) cholesteryl-esters. **B.** Sterols (peaks before 6 min) and unidentified steryl-esters detected in serum pool samples. Note signal intensity of both plant sterols and plant steryl-esters in sitosterolemic serum compared to control.
Lines show XIC traces of the quantifier transitions. Arrow indicate correct campesterol peak. Samples were analyzed using an extended LC-MS/MS method without scheduling.


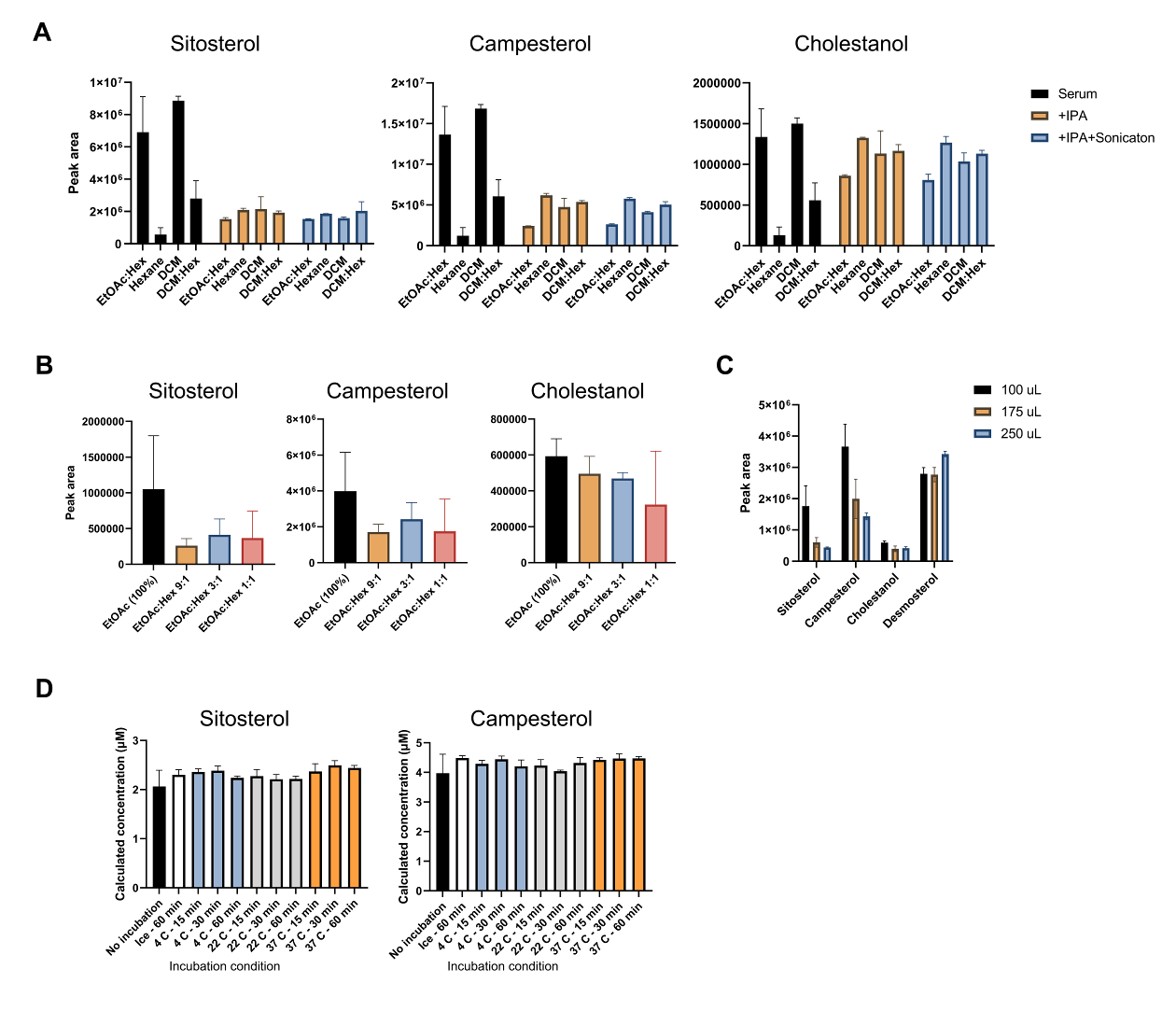


**Figure S8. Sample extraction optimization**
Optimization of sample extraction parameters on sterol and stanol yield (**A** to **D**).
**A**. Effect of sample pre-treatment and extraction solvent. **B**. Optimization of extraction solvent. **C**. Optimization of sample volume. **D**. Effect of incubation time and temperature on absolute quantitation (“LCAT activity”).

## **Supplemental Tables**

**Table S1 Sterol concentrations and fractions reported in literature**

| **Sterol** | **Group** | **Free (µM) ^1^** | **Ester (µM)** | **Total (µM) ^1^** | **Free fraction^2^** | **Reference** |
| --- | --- | --- | --- | --- | --- | --- |
| Sitosterol | Control (n=22) | NA | NA | 5.3 | NA | Salen et al. (14) |
|  | Control (n=49) | 2.0 (0.8 - 5.5) | 3.8 | 5.9 | 35.0% | Lembcke et al. (15) |
|  | Control (n=34) | NA | NA | 4.2 | NA | de Sain-van der Velden et al. (10) |
|  | Parenteral nutrition (adults) | 15.0 | 18.1 | 33.3 | 44.9% | Savini et al. (16) |
|  | Sitosterolemia (n=8) | NA | NA | 590.8 (313.5 – 1350.3) | NA | Salen et al. (14) |
|  | Sitosterolemia (n=12) | 192.9 | 260.4 | 434.0 | 44.4% | Lin et al. (17) |
|  | Sitosterolemia (P1) | 289.4 | 364.1 | 653.5 | 44.3% | Bhattacharyya and Connor (18) |
|  | Sitosterolemia (P2) | 176.0 | 250.8 | 426.8 | 41.2% |  |
|  | CTX (P1) | NA | NA | 16.5 | NA | de Sain-van der Velden et al. (10) |
|  | CTX (P2) | NA | NA | 15.9 | NA |  |
| Campesterol | Control (n=20) | NA | NA | ND | NA | Salen et al. (14) |
|  | Control (n=49) | 3.9 (1.4 - 11.8) | 7.4 | 11.3 | 34.7% | Lembcke et al. (15) |
|  | Control (n=34) | NA | NA | 11.9 | NA | de Sain-van der Velden et al. (10) |
|  | Parenteral nutrition (adults) | 5.2 | 6.7 | 12.0 | 43.8% | Savini et al. (16) |
|  | Sitosterolemia (n=8) | NA | NA | 305.7 (187.2 – 599.0) | NA | Salen et al. (14) |
|  | Sitosterolemia (n=12) | 89.8 | 137.3 | 227.1 | 39.6% | Lin et al. (17) |
|  | Sitosterolemia (P1) | 99.8 | 142.3 | 242.1 | 41.2% | Bhattacharyya and Connor (18) |
|  | Sitosterolemia (P2) | 77.4 | 124.8 | 204.7 | 37.8% |  |
|  | CTX (P1) | NA | NA | 9.7 | NA | de Sain-van der Velden et al. (10) |
|  | CTX (P2) | NA | NA | 8.6 | NA |  |
| Stigmasterol | Control (n=49) | 1.0 (0.4 - 3.0) | 2.4 | 3.3 | 29.2% | Lembcke et al. (15) |
|  | Parenteral nutrition (adults) | 4.1 | 1.2 | 5.3 | 77.3% | Savini et al. (16) |
|  | Sitosterolemia (n=12) | 29.1 | 9.7 | 38.8 | 75.0% | Lin et al. (17) |
|  | Sitosterolemia (P1) | 12.1 | 0.0 | 12.1 | 100.0% | Bhattacharyya and Connor (18) |
|  | Sitosterolemia (P2) | 12.1 | 0.0 | 12.1 | 100.0% |  |
| Desmosterol | Control (n=34) | NA | NA | 2.63 | NA | de Sain-van der Velden et al. (10) |
|  | CTX (P1) | NA | NA | 0.87 | NA |  |
|  | CTX (P2) | NA | NA | 0.63 | NA |  |
|  | Diverse | NA (0.14 - 0.94) | NA (0.39 -1.95) | NA (0.53 – 2.89) | 26.1 – 32.4%^3^ | Bjorkhem et al. (19) |
| 7DHC | Control (n=6) | NA | NA | 0.04 (ND - 0.04) | NA | Ruan et al. (20) |
|  | Control (n=34) | NA | NA | 0.32 | NA | de Sain-van der Velden et al. (10) |
|  | Control (<2 years, n=52) | NA | NA | 0.03 (0.01 - 0.08) | NA | Kelley (21) |
|  | Control (>12 years, n=11) | NA | NA | 0.03 (0.02 - 0.04) | NA |  |
|  | SLOS (n=14) | NA | NA | 25.36 (1.66 - 78.77) | ~ 50% | Ruan et al. (20) |
|  | SLOS (n=35) | NA | NA | 38.48 (0.70 - 122.19) | NA | Kelley (21) |
|  | SLOS (children, n=4) | 124.79 | 197.59 | 322.38 | 38.7% | Lin et al. (17) |
|  | CTX (P1) | NA | NA | 34.29 | NA | de Sain-van der Velden et al. (10) |
|  | CTX (P2) | NA | NA | 71.96 | NA |  |
| 8DHC | Control (n=6) | NA | NA | 0.07 (ND - 0.14) | NA | Ruan et al. (20) |
|  | Control (n=34) | NA | NA | 5.71 | NA | de Sain-van der Velden et al. (10) |
|  | SLOS (n=14) | NA | NA | 18.13 (2.18 - 59.02) | ~ 80 % | Ruan et al. (20) |
|  | CTX (P1) | NA | NA | 43.13 | NA | de Sain-van der Velden et al. (10) |
|  | CTX (P2) | NA | NA | 50.17 | NA |  |
| Lathosterol | Control (n=6) | NA | NA | 0.8 (0.3 - 1.4) | NA | Ruan et al. (20) |
|  | Control (n=34) | NA | NA | 5.0 | NA | de Sain-van der Velden et al. (10) |
|  | SLOS (n=14) | NA | NA | 0.6 (ND - 1.4) | NA | Ruan et al. (20) |
|  | CTX (P1) | NA | NA | 12.6 | NA | de Sain-van der Velden et al. (10) |
|  | CTX (P2) | NA | NA | 12.5 | NA |  |
|  | Diverse | NA (0.4 – 11.9) | NA (0.2 – 8.9) | NA (0.9 – 20.8) | NA 44.5 – 57.2%^3^ | Bjorkhem et al. (19) |
| Cholestanol | Control | NA | NA | NA | 25 – 30%^4^ | Salen and Grundy (22) |
|  | Control | NA | NA | < 12.9 | NA | Duell et al. (23) |
|  | Control | NA | NA | 23.2 | NA | Salen (24) |
|  | Control (n=6) | NA | NA | 0.9 (0.6 - 1.5) | NA | Ruan et al. (20) |
|  | Control (n=34) | NA | NA | 4.9 | NA | de Sain-van der Velden et al. (10) |
|  | Control (n=20) | NA | NA | 5.1 | NA | Salen et al. (14) |
|  | Sitosterolemia (n=8) | NA | NA | 117.4 (41.2 – 283.0) | NA | Salen et al. (14) |
|  | Sitosterolemia (n=12) | 43.7 | 69.5 | 115.8 | 37.8% | Lin et al. (17) |
|  | SLOS (n=14) | NA | NA | 0.5 (ND - 1.1) | NA | Ruan et al. (20) |
|  | CTX | NA | NA | NA | 25 – 30%^4^ | Salen and Grundy (22) |
|  | CTX (n=7) | NA | NA | 54.0 | NA | Salen (24) |
|  | CTX (n=43) | NA | NA | 82.3 (23.2 - 259.9) | NA | Duell et al. (23) |
|  | CTX (P1) | NA | NA | 82.3 | NA | Skrede and Stokke (25) |
|  | CTX (P1) | NA | NA | 30.1 | NA | de Sain-van der Velden et al. (10) |
|  | CTX (P2) | NA | NA | 77.2 | NA | Skrede and Stokke (25) |
|  | CTX (P2) | NA | NA | 27.8 | NA | de Sain-van der Velden et al. (10) |
| Cholesterol | Parenteral nutrition (adults) | 1341.5 | 2442.3 | 3783.8 | 35.5% | Savini et al. (16) |
|  | Sitosterolemia (n=12) | 1838.9 | 3814.8 | 5653.7 | 32.5% | Lin et al. (17) |
|  | Sitosterolemia (P1) | 1453.5 | 3545.8 | 5001.9 | 29.1% | Bhattacharyya and Connor (18) |
|  | Sitosterolemia (P2) | 1282.8 | 4037.2 | 5320.1 | 24.1% |  |
|  | SLOS (children, n=4) | 271.6 | 1686.3 | 1960.4 | 13.9% | Lin et al. (17) |

1: Range given in parenthesis

2: Percentage of free to total sterol concentration (calculated) or reported in the reference.
3: Calculated from min and max of the reported ranges.

4: Intravenous infusion of isotopically-labelled compound.
NA: Not available, ND: Not detected.

**Table S2 Standards and relevant disease**

| **Compound** | **Brand/producer** | **Catalogue no. (quality)** | **Disease** |
| --- | --- | --- | --- |
| Desmosterol | Avanti Polar Lipids | 700060 (> 99%) | Desmosteroliosis |
| d6-Desmosterol | Avanti Polar Lipids | 700040 (> 99%) |  |
| 7-Dehydrocholesterol | Sigma-Aldrich | 30800 (≥ 95%) | Smith-Lemli-Opitz syndrome (SLOS) |
| d7-7-Dehydrocholesterol | Avanti Polar Lipids | 700116 (> 99%) |  |
| Lathosterol | Avanti Polar Lipids | 700069 (> 99%) | Lathosteroliosis |
| Cholesterol | Sigma-Aldrich | C8667 (> 99%) | (Familial hypercholesterolemia) |
| Cholestanol | Supelco | 47129 (CRM-100) | Cerebrotendinous xanthomatosis (CTX) |
| d5-Cholestanol | Avanti Polar Lipids | 700094 (> 99%) |  |
| Campesterol | Avanti Polar Lipids | 700126 (> 99%) | Sitosterolemia |
| d6-Campesterol | Avanti Polar Lipids | 700092 (> 99%) |  |
| Stigmasterol | Supelco | 47132 (CRM-100) |  |
| d5-Stigmasterol | Avanti Polar Lipids | 700157 (> 99%) |  |
| Sitosterol | Supelco | 47133 (CRM-100) |  |
| d7-Sitosterol | Avanti Polar Lipids | 700148 (> 99%) |  |

CRM-100: Certified reference material, quality level 100.

**Table S3. Mass Spectrometer parameters (Compound)**

| **Analyte** | **Type** | **Q1 (m/z)** | **Q3 (m/z)** | **DP (V)** | **EP (V)** | **CE (a.u.)** |
| --- | --- | --- | --- | --- | --- | --- |
| Desmosterol | Quantifier | 367.2 | 161.2 | 60 | 10 | 30 |
| Desmosterol | Qual 1 | 367.2 | 147.3 | 60 | 10 | 34 |
| Desmosterol | Qual 2 | 367.2 | 91.2 | 60 | 10 | 91 |
| Desmosterol | Qual 3 | 367.2 | 105.1 | 60 | 10 | 70 |
| Desmosterol | Qual 4 | 367.2 | 94.9 | 60 | 10 | 45 |
| Desmosterol | Int Standard | 373.4 | 161.1 | 70 | 10 | 32 |
| 7DHC | Quantifier | 367.2 | 91.2 | 60 | 10 | 91 |
| 7DHC | Qual 1 | 367.2 | 105.1 | 60 | 10 | 70 |
| 7DHC | Qual 2 | 367.2 | 147.3 | 60 | 10 | 34 |
| 7DHC | Qual 3 | 367.2 | 94.9 | 60 | 10 | 45 |
| 7DHC | Int Standard | 374.4 | 147.2 | 70 | 10 | 35 |
| Lathosterol^1^ | Quantifier | 369.3 | 301.2 | 70 | 10 | 21 |
| Lathosterol^1^ | Qual 1 | 369.3 | 95.0 | 60 | 10 | 50 |
| Lathosterol^1^ | Qual 2 | 369.3 | 215.2 | 60 | 10 | 28 |
| Lathosterol^1^ | Qual 3 | 369.3 | 161.1 | 60 | 10 | 32 |
| Cholesterol | Quantifier | 369.3 | 161.1 | 70 | 10 | 32 |
| Cholesterol | Qual 1 | 369.3 | 105.2 | 70 | 10 | 60 |
| Cholesterol | Qual 2 | 369.3 | 135.1 | 70 | 10 | 34 |
| Cholestanol | Quantifier | 371.1 | 135.3 | 60 | 10 | 30 |
| Cholestanol | Qual 1 | 371.1 | 91.2 | 60 | 10 | 90 |
| Cholestanol | Qual 2 | 371.1 | 105.1 | 60 | 10 | 65 |
| Cholestanol | Qual 3 | 371.1 | 161.2 | 60 | 10 | 33 |
| Cholestanol | Int Standard | 376.4 | 95.0 | 70 | 10 | 40 |
| Campesterol | Quantifier | 383.3 | 147.2 | 60 | 10 | 36 |
| Campesterol | Qual 1 | 383.3 | 161.3 | 60 | 10 | 33 |
| Campesterol | Qual 2 | 383.3 | 91.0 | 60 | 10 | 88 |
| Campesterol | Qual 3 | 383.3 | 104.9 | 60 | 10 | 66 |
| Campesterol | Int Standard | 389.4 | 147.1 | 70 | 10 | 37 |
| Stigmasterol | Quantifier | 395.2 | 297.3 | 60 | 10 | 25 |
| Stigmasterol | Qual 1 | 395.2 | 161.2 | 60 | 10 | 33 |
| Stigmasterol | Qual 2 | 395.2 | 147.2 | 60 | 10 | 30 |
| Stigmasterol | Qual 3 | 395.2 | 255.2 | 60 | 10 | 23 |
| Stigmasterol | Int Standard | 400.4 | 147.0 | 70 | 10 | 38 |
| Sitosterol | Quantifier | 397.1 | 147.2 | 70 | 10 | 35 |
| Sitosterol | Qual 1 | 397.1 | 161.4 | 70 | 10 | 30 |
| Sitosterol | Qual 2 | 397.1 | 105.0 | 70 | 10 | 73 |
| Sitosterol | Qual 3 | 397.1 | 91.0 | 70 | 10 | 85 |
| Sitosterol | Int Standard | 404.4 | 147.2 | 60 | 10 | 37 |

1. The Quantifier is the only useful transition to measure lathosterol in serum with the reported regular chromatographic method (due to high cholesterol concentration).
Abbreviations: Q1: first quadrupole/precursor, Q3: third quadrupole/fragment, DP: declustering potential, EP: entrance potential. CE: collision energy, a.u.: arbitrary units, Qual: qualifier transition.

**Table S4. Mass Spectrometer parameters – Source/Gas**

| **Setting** | **Value** | **Unit** |
| --- | --- | --- |
| Curtain gas (CUR) | 45 | a.u. |
| Collision gas (CAD) | Medium | - |
| IonSpray Voltage (IS) | 5500 | V |
| Temperature (TEM) | 500 | ° C |
| Ion Source Gas 1 (GS1) | 60 | a.u. |
| Ion Source Gas 2 (GS2) | 60 | a.u. |
| Electrospray ionization polarity | Positive (+) | - |

a.u. arbitrary units

**Table S5 Sterol concentrations in calibrator stocks (“2x stocks”)**

| **Analyte** | **Cal 10** | **Cal 9** | **Cal 8** | **Cal 7** | **Cal 6** | **Cal 5** | **Cal 4** | **Cal 3** | **Cal 2** | **Cal 1** |
| --- | --- | --- | --- | --- | --- | --- | --- | --- | --- | --- |
| Desmosterol | 20 | 15 | 10 | 5 | 2 | 1 | 0.5 | 0.25 | 0.1 | 0.05 |
| 7DHC | 40 | 30 | 20 | 10 | 4 | 2 | 1 | 0.5 | 0.2 | 0.1 |
| Lathosterol | 10 | 7.5 | 5 | 2.5 | 1 | 0.5 | 0.25 | 0.125 | 0.05 | 0.025 |
| Cholesterol | 40 | 30 | 20 | 10 | 4 | 2 | 1 | 0.5 | 0.2 | 0.1 |
| Cholestanol | 40 | 30 | 20 | 10 | 4 | 2 | 1 | 0.5 | 0.2 | 0.1 |
| Campesterol | 40 | 30 | 20 | 10 | 4 | 2 | 1 | 0.5 | 0.2 | 0.1 |
| Stigmasterol | 40 | 30 | 20 | 10 | 4 | 2 | 1 | 0.5 | 0.2 | 0.1 |
| Sitosterol | 40 | 30 | 20 | 10 | 4 | 2 | 1 | 0.5 | 0.2 | 0.1 |

All concentration values are in µM. Dissolved in isopropanol.

**Table S6 Method validation parameters – Quantitation Limits**

| Analyte | Disease marker | OMIM no. (Orpha)^1^ | Quantitative | LLOQ^2^ (µM) | mLLOQ ^2^ (µM) | mULOQ^2^ (µM) | Bias in undiluted pathological serum^4^ |
| --- | --- | --- | --- | --- | --- | --- | --- |
| Desmosterol | Desmosterolosis | 602398 (35107) | Yes | 0.025 | 0.125 | 10 | NP |
| 7-Dehydrocholesterol (7DHC)^5^ | SLOS | 270400 (818) | Yes^5^ | 0.05 | 5.7 | 20 | - 33% |
| Lathosterol^6^ | Lathosterolosis | 607330 (46059) | Semi^6^ | 0. 125 | NA | 5 | NP |
| Cholestanol | CTX | 213700 (909) | Yes | 0.25 | 0.25 | 15 | - 15 % |
| Campesterol | Sitosterolemia | 210250 / 618666 (2882) | Yes | 0.05 | 0.25 | 20 | - 13 % |
| Stigmasterol^5^ |  |  | Yes^5^ | 0.1 | 1.5 | 15 | - 4.4 % |
| Sitosterol |  |  | Yes | 0.05 | 0.25 | 15 | 5.4 % |

1. OMIM: Online Mendelian Inheritance in Man identifier; ORPHA: Orphanet Rare Disease Ontology identifier.
2. LLOQ: Lowest level of quantitation validated for sterol standards in LD-serum. mLLOQ: Method lower level of quantitation (pragmatic) due to interferences present in normal (undiluted) serum. mULOQ: Method upper level of quantitation of undiluted serum (no extrapolation)
3. Accuracy calculated from the (measured) difference in concentration between serum pool and pool spiked with 10 µM of analytes (desmosterol, lathosterol, cholestanol, campesterol and sitosterol) or by the measured increase in concentration between serum spiked with 2 and 10 µM (7-dehydrocholesterol and stigmasterol) sterols.
4. Estimated bias for quantitation in undiluted serum (sitosterolemia or SLOS-patients) by extrapolation of the linear calibration curves.

NA: not applicable, NP: not performed

1. 7-Dehydrocholesterol and stigmasterol concentrations were too low for quantitation in normal serum. 7DHC co-measured presumed 8-dehydrocholesterol (also elevated in SLOS). Increase of 2 µM 7DHC (spiking) was qualitatively apparent.
2. Lathosterol is semi-quantitated using d7-7DHC.

**Table S7 Method validation parameters – Imprecision, Matrix Effects and Absolute Recovery**

|  | Imprecision, intraassay (%CV) | | | | | Imprecision, interassay (%CV) | | | Matrix effects | Absolute Recovery |
| --- | --- | --- | --- | --- | --- | --- | --- | --- | --- | --- |
|  | **Control** | **Sitosterolemia** | | **SLOS** | | **Control** | **Sitosterolemia** | |  |  |
| Analyte |  | **Diluted^1^** | **Undiluted^2^** | **Diluted^1^** | **Undiluted^2^** |  | **Diluted^1^** | **Undil^2^** |  |  |
| Desmosterol | 4.0 % | NA | 2.9 % | NP | NP | 5.7 % | NA | 2.3 % | - 4 % | 26.1 % |
| 7-DHC^4,5^ | 5.5 %^4^ | NA | NP | 7.1% | 2.6% | 1.0 %^5^ | NA | NP | - 13 % | 24.1 % |
| Lathosterol | 6.0 % | NA | NP | NP | NP | 6.9 % | NA | NP | NA | NP |
| Cholestanol | 5.0 % | NA | 3.7 % | NP | NP | 9.5 % | NA | 2.9 % | - 26 % | 24.8 % |
| Campesterol | 2.1 % | 2.8 % | 4.0 % | NP | NP | 7.7 % | 8.3 % | 3.4% | - 27 % | 23.3 % |
| Stigmasterol^4,5^ | 5.1 %^4^ | 6.0 % | 3.8 % | NP | NP | 4.7 %^5^ | 1.2% | 10.0 % | - 28 % | 26.7 % |
| Sitosterol | 2.7 % | 1.5% | 1.7 % | NP | NP | 3.9 % | 8.0 % | 1.0 % | - 25 % | 21.0 % |

1. Imprecision for patient serum diluted using LD-serum to be within quantitative range.
2. Imprecision for undiluted patient serum. The sterols campesterol, sitosterol (sitosterolemia) or 7-dehydrocholesterol (SLOS) were above mULOQ and the calibrators extrapolated linearly.
3. Estimated bias for quantitated sterol concentration in undiluted, pathological samples (using extrapolated calibrators) compared to results after dilution to within calibrator range. 7DHC measured using SLOS patient serum, plant sterols using sitosterolemia-pool.
4. Intraday %CV was calculated from control serum pool spiked with 2 µM of stigmasterol and 7DHC.
5. Interday %CV was calculated from control serum pool spiked with 1.5 µM or 4.0 µM stigmasterol or 7DHC, respectively.

**Table S8 Accuracy of measured increase (“spike and recover”)**

|  | **Spike level** | | |
| --- | --- | --- | --- |
| **Analyte** | **Low** | **Moderate** | **High** |
| Desmosterol^1^ | 107 % | 107 % | 99 % |
| 7DHC^2^ | NA | 113 % | 99 % |
| Lathosterol^1,3^ | 76 % | 75 % | 64 % |
| Cholestanol^1^ | 102 % | 103 % | 92 % |
| Campesterol^1^ | 105 % | 102 % | 96 % |
| Stigmasterol^2^ | NA | 115 % | 102 % |
| Sitosterol^1^ | 123 % | 119 % | 109 % |

1. Measured increase for serum spiked with 2 (Low), 5 (Medium) or 10 µM (High) of each standard compared to non-spiked control.
2. Measured increase compared to serum spiked with 2 µM. Relative increase of 3 (Medium) or 8 µM (High).
3. Lathosterol is only semi-quantitative.
   NA: Not applicable, non-spiked control not quantitatable.

**Table S9 Accuracy Comparison to ERNDIM IQCS Special Assays in Serum Controls**

|  | **7DHC** | | **Cholestanol** | |
| --- | --- | --- | --- | --- |
| **Concentration level** | **Target, µM^1^** | **Measured, µM^2^ (Accuracy)** | **Target, µM^1^** | **Measured, µM^2^ (Accuracy)** |
| Medium (L1) | 16 | 18.8 (118%) | 21 | 15.7 (75%) |
| High (L2) | 80 | 85.5 (107%) | 66 | 61.0 (92%) |

1. Concentration (estimated) provided on ERNDIM product certificate. ERNDIM SAS-02.1 and SAS-02.2, batch 2020.1321 and 2020.1322. Date 15 Oct 2020.
2. Mean concentration of a minimum of four replicates split between at least two batches.

## **Supplemental References**

1. Liu W, Xu L, Lamberson CR, Merkens LS, Steiner RD, Elias ER, et al. Assays of plasma dehydrocholesteryl esters and oxysterols from Smith-Lemli-Opitz syndrome patients. J Lipid Res 2013 Jan;54:1:244-53 as doi: 10.1194/jlr.M031732.

2. Xu L, Korade Z, Porter NA. Oxysterols from free radical chain oxidation of 7-dehydrocholesterol: product and mechanistic studies. J Am Chem Soc 2010 Feb 24;132:7:2222-32 as doi: 10.1021/ja9080265.

3. Gardner MS, McWilliams LG, Jones JI, Kuklenyik Z, Pirkle JL, Barr JR. Simultaneous Quantification of Free Cholesterol, Cholesteryl Esters, and Triglycerides without Ester Hydrolysis by UHPLC Separation and In-Source Collision Induced Dissociation Coupled MS/MS. J Am Soc Mass Spectrom 2017 Nov;28:11:2319-29 as doi: 10.1007/s13361-017-1756-2.

4. Acimovic J, Lövgren-Sandblom A, Monostory K, Rozman D, Golicnik M, Lütjohann D, Björkhem I. Combined gas chromatographic/mass spectrometric analysis of cholesterol precursors and plant sterols in cultured cells. J Chromatogr B Analyt Technol Biomed Life Sci 2009 Jul 15;877:22:2081-6 as doi: 10.1016/j.jchromb.2009.05.050.

5. R Core Team. R: A Language and Environment for Statistical Computing. Vienna, Austria: R Foundation for Statistical Computing; 2023.

6. RStudio Team. RStudio: Integrated Development for R. Boston, MA, USA: RStudio; 2020.

7. Hothorn T, Hornik K, van de Wiel MA, Zeileis A. Implementing a class of permutation tests: The coin package. Journal of Statistical Software 2008;28:8:1-23 as doi: doi:10.18637/jss.v028.i08.

8. Wickham H, Averick M, Bryan J, Chang W, McGowan L, Françoi R, et al. Welcome to the Tidyverse. Journal of Open Source Software 2019;4:43:1686 as doi: 10.21105/joss.01686.

9. Kunnen S, Van Eck M. Lecithin:cholesterol acyltransferase: old friend or foe in atherosclerosis? J Lipid Res 2012 Sep;53:9:1783-99 as doi: 10.1194/jlr.R024513.

10. de Sain-van der Velden MG, Verrips A, Prinsen BH, de Barse M, Berger R, Visser G. Elevated cholesterol precursors other than cholestanol can also be a hallmark for CTX. J Inherit Metab Dis 2008 Dec;31 Suppl 2:S387-93 as doi: 10.1007/s10545-008-0963-1.

11. Griffiths WJ, Abdel-Khalik J, Crick PJ, Ogundare M, Shackleton CH, Tuschl K, et al. Sterols and oxysterols in plasma from Smith-Lemli-Opitz syndrome patients. J Steroid Biochem Mol Biol 2017 May;169:77-87 as doi: 10.1016/j.jsbmb.2016.03.018.

12. McDonald JG, Smith DD, Stiles AR, Russell DW. A comprehensive method for extraction and quantitative analysis of sterols and secosteroids from human plasma. J Lipid Res 2012 Jul;53:7:1399-409 as doi: 10.1194/jlr.D022285.

13. Vladimirov S, Gojkovic T, Zeljkovic A, Jelic-Ivanovic Z, Spasojevic-Kalimanovska V. Determination of non-cholesterol sterols in serum and HDL fraction by LC/MS-MS: Significance of matrix-related interferences. J Med Biochem 2020 Sep 2;39:3:299-308 as doi: 10.2478/jomb-2019-0044.

14. Salen G, Shefer S, Nguyen L, Ness GC, Tint GS, Shore V. Sitosterolemia. J Lipid Res 1992 Jul;33:7:945-55.

15. Lembcke J, Ceglarek U, Fiedler GM, Baumann S, Leichtle A, Thiery J. Rapid quantification of free and esterified phytosterols in human serum using APPI-LC-MS/MS. J Lipid Res 2005 Jan;46:1:21-6 as doi: 10.1194/jlr.C400004-JLR200.

16. Savini S, Correani A, Pupillo D, D'Ascenzo R, Biagetti C, Pompilio A, et al. Phytosterol Esterification is Markedly Decreased in Preterm Infants Receiving Routine Parenteral Nutrition. Lipids 2016 Dec;51:12:1353-61 as doi: 10.1007/s11745-016-4197-y.

17. Lin DS, Steiner RD, Merkens LS, Pappu AS, Connor WE. The effects of sterol structure upon sterol esterification. Atherosclerosis 2010 Jan;208:1:155-60 as doi: 10.1016/j.atherosclerosis.2009.07.031.

18. Bhattacharyya AK, Connor WE. Beta-sitosterolemia and xanthomatosis. A newly described lipid storage disease in two sisters. J Clin Invest 1974 Apr;53:4:1033-43 as doi: 10.1172/JCI107640.

19. Bjorkhem I, Miettinen T, Reihner E, Ewerth S, Angelin B, Einarsson K. Correlation between serum levels of some cholesterol precursors and activity of HMG-CoA reductase in human liver. J Lipid Res 1987 Oct;28:10:1137-43.

20. Ruan B, Wilson WK, Pang J, Gerst N, Pinkerton FD, Tsai J, et al. Sterols in blood of normal and Smith-Lemli-Opitz subjects. J Lipid Res 2001 May;42:5:799-812.

21. Kelley RI. Diagnosis of Smith-Lemli-Opitz syndrome by gas chromatography/mass spectrometry of 7-dehydrocholesterol in plasma, amniotic fluid and cultured skin fibroblasts. Clin Chim Acta 1995 Apr 30;236:1:45-58 as doi: 10.1016/0009-8981(95)06038-4.

22. Salen G, Grundy SM. The metabolism of cholestanol, cholesterol, and bile acids in cerebrotendinous xanthomatosis. J Clin Invest 1973 Nov;52:11:2822-35 as doi: 10.1172/JCI107478.

23. Duell PB, Salen G, Eichler FS, DeBarber AE, Connor SL, Casaday L, et al. Diagnosis, treatment, and clinical outcomes in 43 cases with cerebrotendinous xanthomatosis. J Clin Lipidol 2018 Sep-Oct;12:5:1169-78 as doi: 10.1016/j.jacl.2018.06.008.

24. Salen G. Cholestanol deposition in cerebrotendinous xanthomatosis. A possible mechanism. Ann Intern Med 1971 Dec;75:6:843-51 as doi: 10.7326/0003-4819-75-6-843.

25. Skrede S, Stokke KT. Plasma esterification of cholestanol, normally and in cerebrotendinous xanthomatosis. Scand J Clin Lab Invest 1974 Apr;33:2:97-100.
